# Double role of metalloporphyrins in catalytic bioinspired supramolecular metal–organic frameworks (SMOFs)

**DOI:** 10.1107/S2052252518007856

**Published:** 2018-07-20

**Authors:** Arkaitz Fidalgo-Marijuan, Eder Amayuelas, Gotzone Barandika, Edurne S. Larrea, Begoña Bazán, Miren Karmele Urtiaga, Marta Iglesias, María Isabel Arriortua

**Affiliations:** a Mineralogía y Petrología, Universidad del País Vasco (UPV/EHU), Barrio Sarriena s/n, Leioa, Bizkaia 48940, Spain; b BCMaterials, Basque Center for Materials, Applications and Nanostructures, Bld. Martina Casiano, 3rd Floor, UPV/EHU Science Park, Barrio Sarriena s/n, Leioa, Bizkaia 48940, Spain; c Química Inorgánica, Universidad del País Vasco (UPV/EHU), Barrio Sarriena s/n, Leioa, Bizkaia 48940, Spain; d Materials Science Factory, Instituto de Ciencia de Materiales de Madrid-CSIC, Sor Juana Inés de la Cruz 3, Cantoblanco, Madrid 28049, Spain

**Keywords:** metalloporphyrins, supramolecular MOFs, heterogeneous catalysts, Knoevenagel condensations, aldol condensations, one-pot cascade reactions

## Abstract

Heterogeneous catalysts are of great interest in many industrial processes for environmental reasons and, during recent years, a great effort has been devoted to obtain metal–organic frameworks (MOFs) with improved catalytic behaviour. Few supramolecular metal–organic frameworks (SMOFs) are stable under ambient conditions and those with anchored catalysts exhibit favourable properties. However, this paper presents an innovative approach that consists of using metal nodes as both structural synthons and catalysts. Regarding the latter, metalloporphyrins are suitable candidates to play both roles simultaneously. In fact, there are a number of papers that report coordination compounds based on metalloporphyrins exhibiting these features. Thus, the aim of this bioinspired work was to obtain stable SMOFs (at room temperature) based on metallo­porphyrins and explore their catalytic activity. This work reports the environmentally friendly microwave-assisted synthesis and characterization of the compound [H(bipy)]_2_[(MnTPPS)(H_2_O)_2_]·2bipy·14H_2_O (TPPS = *meso*-tetra­phenyl­porphine-4,4′,4′′,4′′′-tetra­sulfonic acid and bipy = 4,4′-bi­pyridine). This compound is the first example of an MnTPPS-based SMOF, as far as we are aware, and has been structurally and thermally characterized through single-crystal X-ray diffraction, IR spectroscopy, thermogravimetry and transmission electron microscopy. Additionally, this work explores not only the catalytic activity of this compound but also of the compounds μ-*O*-[FeTCPP]_2_·16DMF and [CoTPPS_0.5_(bipy)(H_2_O)_2_]·6H_2_O. The structural features of these supra­molecular materials, with accessible networks and high thermal stability, are responsible for their excellent behaviour as heterogeneous catalysts for different oxidation, condensation (aldol and Knoevenagel) and one-pot cascade reactions.

## Abstract 2

Bioinspired metalloporphyrin-based supramolecular MOFs have been successfully tested for heterogeneous catalytic applications.

## 1. Introduction   

During recent years, supramolecular materials and metal–organic frameworks (MOFs) have been thoroughly explored in many fields, such as water reuse, photocatalysis, electrochemistry and gas adsorption (de Lange *et al.*, 2015 ▸; Dias & Petit, 2015 ▸; Li *et al.*, 2016 ▸; Li & Hill, 2017 ▸; Wang *et al.*, 2016 ▸; Gao *et al.*, 2014 ▸). Their structural and chemical properties make them excellent candidates as solid catalysts for many reactions (Dhakshinamoorthy *et al.*, 2012 ▸). Moreover, supra­molecular metal–organic frameworks (SMOFs), in which the three-dimensional crystalline network is sustained by hydrogen bonds (Pérez-Aguirre *et al.*, 2016 ▸; Reger *et al.*, 2012 ▸; Thomas-Gipson *et al.*, 2014 ▸), are attracting much interest, and in order to obtain those coordination networks, the use of porphyrins has been rising, since they are organic ligands which present unique properties attached to biochemical, enzymatic and photochemical functions (Kornienko *et al.*, 2015 ▸; Spoerke *et al.*, 2017 ▸). Biomimetic catalysts, such as metalloporphyrins, have been used as cytochrome analogues because of the similarity between these molecules and the active centres of the enzymes. Oxidation, condensation and hydrolysis reactions are very common in living organisms and many efforts have been made to mimic their catalytic activity by means of metalloporphyrin-based synthetic models (Johnson *et al.*, 2016 ▸; Chen *et al.*, 2012 ▸; Feng *et al.*, 2012 ▸). In fact, metalloporphyrinic supramolecular compounds are appearing as a new class of promising materials in the development of catalytic cascade or one-pot reactions (Hajimohammadi *et al.*, 2012 ▸; Shinde *et al.*, 2015 ▸; Prasad *et al.*, 2014 ▸; Lu *et al.*, 2015 ▸). Most of the catalytic reactions in industry use the traditional design of simple catalytic reactions involving expensive catalysts and processes. So, in order to reduce costs and optimize processes, the majority of recent work has focused on homogeneous (Omagari *et al.*, 2016 ▸; Bonin *et al.*, 2014 ▸; Costentin *et al.*, 2014 ▸; Pires *et al.*, 2014 ▸) or heterogeneous catalytic activity based on metalloporphyrinic networks (Zhang *et al.*, 2016 ▸; Chen *et al.*, 2015 ▸; Hod *et al.*, 2015 ▸; Ucoski *et al.*, 2015 ▸; Wang *et al.*, 2013 ▸; Meng *et al.*, 2012 ▸). There are some disadvantages of heterogeneous catalysis, including poor understanding of the reaction mechanisms, heat diffusion problems, low reaction selectivity and poorly defined active sites. However, recycling is one of the most important advantages for heterogeneous catalysis and it is based on easy catalyst separation (Moulijn *et al.*, 1993 ▸).

In order to achieve heterogeneous catalysis, there are a number of successful approaches such as anchoring the catalyst into the cavities of porous coordination networks (Zhan & Zeng, 2016 ▸; Liu *et al.*, 2016 ▸), doping the network with the catalyst (Lan *et al.*, 2016 ▸) or post functionalizing the network (Andriamitantsoa *et al.*, 2016 ▸). In this sense, we have been exploring a new strategy that consists of using porphyrins as structural units in SMOFs and catalytic active centres simultaneously (Fidalgo-Marijuan *et al.*, 2015 ▸). This strategy includes the use of first-row transition metals, avoiding commonly used heavy and toxic metals such as Ru, Rh and Ce (Liu *et al.*, 2017 ▸). It is also noteworthy that we have performed green syntheses, using preferably non-toxic solvents (water) and fast microwave heating.

Taking into consideration the above-mentioned aspects, we report here the heterogeneous catalysis of three porphyrinic SMOFs: [H(bipy)]_2_[(MnTPPS)(H_2_O)_2_]·2bipy·14H_2_O, **1**, having MnTPPS-based monomers, the *μ*-*O*-[FeTCPP]_2_·16DMF dimeric compound, **2**, and the [CoTPPS_0.5_(bipy)(H_2_O)_2_]·6H_2_O one-dimensional compound, **3**, where TPPS = *meso*-tetra­phenyl­porphine-4,4′,4′′,4′′′-tetra­sulfonic acid, TCPP = *meso*-tetra(4-carb­oxy­phenyl)­porphine, bipy = 4,4′-bi­pyridine and DMF = *N*,*N*′-di­methyl­formamide. It is worth noting that compound **1** is the first Mn TPPS metallo­porphyrinic SMOF reported so far. We have reported the structural features of **2** and **3** elsewhere (Fidalgo-Marijuan *et al.*, 2015 ▸, Fidalgo-Marijuan *et al.*, 2013 ▸).

Compounds **1**, **2** and **3** have been exhaustively characterized by means of single-crystal X-ray diffraction, IR spectroscopy, thermogravimetric analysis and transmission electron microscopy (TEM), after which oxidation, Knoevenagel and aldol condensations, and a one-pot cascade reaction (involving an acetal hydrolysis in the first step and a Knoevenagel condensation in the second) have been successfully tested for these compounds.

## 2. Experimental   

### 2.1. General   

All solvents and reagents including *meso*-tetra­phenyl­porphine-4,4′,4′′,4′′′-tetra­sulfonic acid tetrasodium salt (TPPS), 4,4′-bi­pyridine (bipy) and Mn(NO_3_)_2_·*x*H_2_O were purchased from Sigma–Aldrich.

### 2.2. Synthesis of [H(bipy)]_2_[(MnTPPS)(H_2_O)_2_]·2bipy·14H_2_O   

TPPS (10.2 mg, 0.01 mmol) and Mn(NO_3_)_2_·*x*H_2_O (1.0 mg, 0.006 mmol) were dissolved in distilled water (10 ml) and the solution was stirred for 30 min. Then, 4,4′-bi­pyridine (9.4 mg, 0.06 mmol) was dissolved in hot (343 K) distilled water (5 ml) and added to the mixture in a 100 ml CEM EasyPrep microwave vessel. The mixture was heated by microwaves under autogenous pressure at 433 K for 2 h, and then cooled naturally to room temperature, yielding diffraction-quality prismatic dark-red crystals. [For C_42_H_46_Mn_0.5_N_6_O_14_S_2_. Found: C, 53.55 (4); H, 4.85 (2); N, 8.84 (6); O, 22.95 (6); S, 6.89 (4). Calculated: C, 53.08; H, 4.88; N, 8.84; O, 23.57; S, 6.75]. *ν*_max_/cm^−1^ 3397 (OH), 1636–1413 (CC), 1393 and 1180 (SO), 1340 (CN), 1204 and 1070 (bipy), 1003 (MnTPPS) and 857–634 (CH) (see Fig. S1 in the supporting information).

### 2.3. Single-crystal X-ray diffraction   

Prismatic dark-red single-crystals of compound **1** (dimensions given in Table 4) were selected under a polarizing microscope and mounted on MicroMounts. Single-crystal X-ray diffraction data were collected at 100 K on a SuperNova single source diffractometer with Cu *K*α radiation (λ = 1.54184 Å). Data frames were processed [unit-cell determination, intensity-data integration, correction for Lorentz and polarization effects (Yinghua, 1987 ▸) and analytical absorption correction] using the *CrysAlisPro* software package (Agilent Technologies UK Ltd, 2012).

The structure of **1** was solved in the triclinic 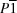 space group using the *SIR92* program (Altomare *et al.*, 1993 ▸) which allowed us to determine the position of the Mn atom, as well as some of the O, N, S and C atoms of the porphyrin and bi­pyridine molecules. The refinement of the crystal structure was performed by full matrix least-squares based on *F* ^2^, using the *SHELXL97* program (Sheldrick, 2008 ▸), obtaining the remaining C, N, O and S atoms of the porphyrin and O atoms of water molecules. Anisotropic displacement parameters (Farrugia, 1997 ▸) were used for all non-hydrogen atoms, except for the disordered crystallized water molecules (Fig. S2). All H atoms connected to aromatic rings (C—H = 0.95 Å) were fixed geometrically and refined using a riding model with common isotropic displacements. Four of the crystallized water mol­ecules of compound **1** were disordered over two groups, with half occupancy each, as well as for one of the porphyrin sulfite groups. Crystal data for compound **1** are listed in Table 1 ▸. Geometric parameters, atomic coordinates and anisotropic displacement parameters are given in the supporting information, Tables S1, S2 and S3.

Diameter values of the channels for compounds **1, 2** and **3** were calculated using the program *TOPOS* (available at http://www.topos.ssu.samara.ru).

### 2.4. Physicochemical characterization techniques   

The IR spectra were collected on a JASCO FT/IR-6100 spectrometer at room temperature in the range 4000–400 cm^−1^, in KBr pellets (1% sample). C, H, N, S and O elemental analyses were measured using a Euro EA 3000 elemental analyser. Thermogravimetric analyses were carried out using a NETZSCH STA 449 F3 thermobalance. A crucible containing approximately 10 mg of sample was heated at 5 K min^−1^ in the temperature range 303–873 K.

TEM work was done on a Philips SuperTwin CM200 operated at 200 kV and equipped with an LaB_6_ filament and EDAX EDS microanalysis system. The samples for TEM analysis were prepared by dispersion into ethanol and keeping the suspension in an ultrasonic bath for 15 min. After that, a drop of the suspension was spread onto a TEM copper grid (300 mesh) covered by a holey carbon film, followed by drying under vacuum.

### 2.5. Catalytic activity   

The oxidation reactions of benzyl alcohol, 1-phenyl­ethanol, 1-hexanol and 1-octanol were carried out at 343 K using aceto­nitrile as the solvent. The catalyst/substrate molar ratio (based on the metal) used for all the reactions was 5:100. Powdered crystals of the catalysts were initially dried at 373 K under vacuum to remove solvent and water adsorbed on the surface.

Before the reactions, approximately 5 mg of dried catalyst was activated by stirring with the oxidizing agent *tert*-butyl hydro­peroxide (TBHP) in 2 ml of aceto­nitrile for 30 min at 343 K. After this activation stage, the catalyst was separated from the liquid medium by centrifugation. The reactor was then charged with the activated catalyst and the corresponding alcohol in 2 ml of solvent. The mixture was heated to 343 K and then the oxidizing agent was added dropwise (1.5 equivalents of TBHP).

Aldol condensation reactions of benzaldehyde, *p*-tolu­aldehyde, *p*-methoxybenzaldehyde and heptanal were carried out at 373 K without solvent. The catalyst/substrate molar ratio (based on the metal) used for all the reactions was 10:100. Powdered crystals of the catalysts were first dried at 373 K under vacuum to remove solvent and water adsorbed on the surface. The reactor was charged with the catalyst (10 mg), acetone (1 ml) and the corresponding substrate, and the mixture was then heated to 373 K.

Knoevenagel condensation reactions of benzaldehyde, *p*-tolu­aldehyde, *p*-fluoro­benzaldehyde, 4-chloro­benzaldehyde and 3-nitro­benzaldehyde were carried out at 343 K using toluene as the solvent. The catalyst/substrate molar ratio (based on the metal) used for all the reactions was 5:100. Powdered crystals of the catalysts were first dried at 373 K under vacuum to remove solvent and water adsorbed on the surface. The reactor was charged with the catalyst (5 mg), malono­nitrile (4.6 mg), do­decane as internal standard (2.0 µl) and the corresponding substrate in 2 ml of solvent, and then the mixture was heated to 343 K.

The one-pot cascade reaction was tested for acetal hydrolysis followed by Knoevenagel condensation at 343 K in toluene. The catalyst/substrate molar ratio (based on the metal) used for the reaction was 10:100. Powdered crystals of the catalysts were first dried at 373 K under vacuum to remove solvent and water adsorbed on the surface. The reactor was charged with the catalyst (5.2 mg), toluene (2 ml), benz­aldehyde di­methyl acetal (5.3 µl), malono­nitrile (2.3 mg) and do­decane (2 µl) as internal standard, and then the mixture was heated to 343 K.

Detailed results for the catalytic activity exhibited by compounds **1**, **2** and **3** will be described in Sections 3.3 and 3.4.

Reaction samples were taken at regular times and analysed on a Hewlett–Packard 5890 II GC–MS or on a Konik HCGC 5000B gas chromatograph–mass spectrometer. Blank experiments were carried out under the reaction conditions in order to determine the extent of the uncatalysed reaction; for all the blank reactions only traces of the product were found. After the reaction, the catalysts were filtered, dried and characterized by IR spectroscopy, and by TEM microscopy in some cases. The calculations of turnover frequencies (TOF; turnover frequency is mol substrate converted/mol catalyst per hour) were performed in the initial stages of the reaction, when the reaction rates are higher, as usual.

## 3. Results and discussion   

### 3.1. Crystal structures   

The structural features of these materials are of great importance in order to achieve satisfactory conversion rates for catalytic reactions. In this sense, the metalloporphyrinic synthons are intended to act as heterogeneous catalysts and structural building units at the same time. In relation to the latter, accessibility of the guest molecules to catalytic metal centres on the surface of the crystal-network cavities is one of the most important features necessary to consider the compounds as potential catalysts, and was taken into account for compounds **1, 2** and **3**.

Compound **1** with the formula [H(bipy)]_2_[(MnTPPS)(H_2_O)_2_]·2bipy·14H_2_O is a coordination compound consisting of complex ions. The crystal structure, determined by single-crystal X-ray diffraction, shows [(MnTPPS)(H_2_O)_2_]^2−^ anionic monomers where TPPS^4−^ ligands are present. The Mn^II^ ion is in an octahedral coordination environment, bonded to the four porphyrin N atoms and with two water molecules in the axial positions.

**Figure 1:**
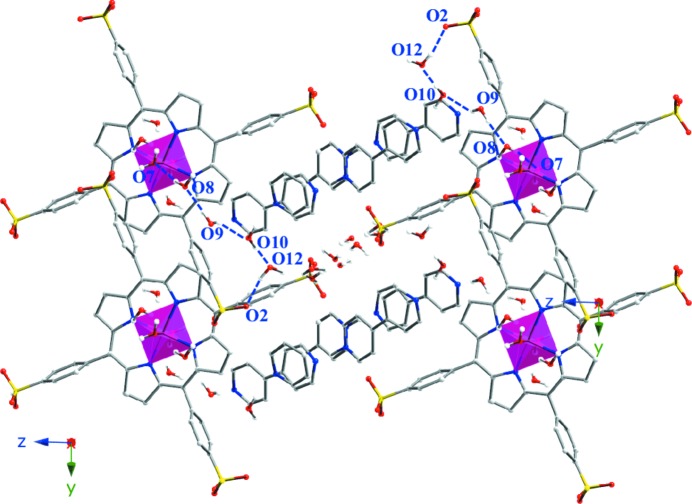
Crystal-structure packing for compound **1**. Colour code: Mn pink, C grey, N blue, O red and S yellow. H atoms have been omitted for clarity.

The [(MnTPPS)(H_2_O)_2_]^2−^ anions crystallize as shown in Fig. 1 ▸. The voids generated between metalloporphyrinic monomers are occupied by [H(bipy)]^+^ cations and crystallized bi­pyridine molecules and, as shown in Fig. S3, those bi­pyridine molecules were pairwise hydrogen-bonded [N5—H1N⋯N4; 2.741 7 Å and 171 9°] and parallel-stacked, giving rise to robust π–π interactions (centroid-to-centroid distances are 3.468 and 3.746 Å) (Soltanzadeh & Morsali, 2009 ▸). Additionally, the interstitial voids are occupied by 14 crystallized water molecules per monomer.

**Figure 2:**
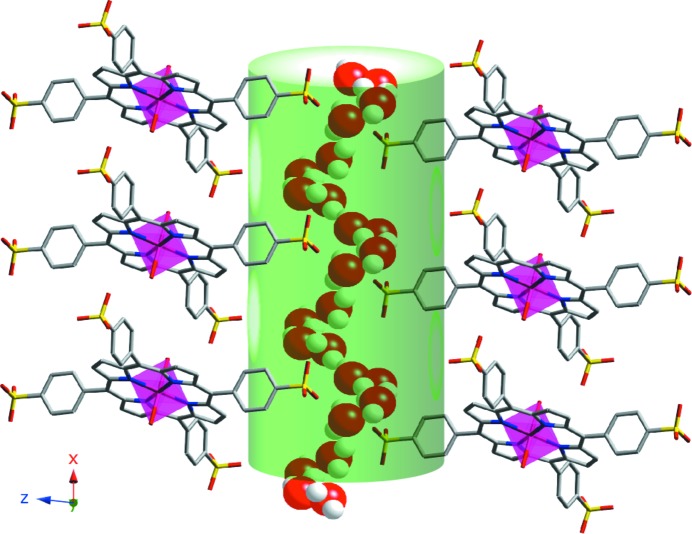
Representation of the accessibility of guest molecules to the active metal centres for compound **1**. Colour code: Mn pink, C grey, N blue, O red and S yellow. The accessible pathway is represented by the green cylinder.

The crystal structure is stabilized by an intricate hydrogen-bonded system (Table S4), connecting the [(MnTPPS)(H_2_O)_2_]^2−^ units along the 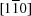 direction between the axial water molecule (O7) and the sulfonate groups (O3) (Fig. S4). Additionally, the metalloporphyrinic monomers are linked by a hydrogen-bonded chain along the [100] direction. This connection involves the coordinated water molecules (O7) and two crystallized water molecules (O8 and O11). It is worth noting that there is a zigzag chain of water molecules along the [100] direction which stabilizes the structure. This chain is located between the sulfonate groups involving the O12 to O16 crystallized water molecules (Fig. 2 ▸).

As shown in Fig. 1 ▸ and Fig. S5, the [H(bipy)_2_]^+^ cations are located on the interporphyrinic voids and linked through O9, O10 and O12 water molecules to the sulfonate groups (O2) and through O8 to the axial water molecules (O7).

Fig. 2 ▸ shows the accessibility of the crystal structure to external guest molecules in order to access channels along the *x* axis. The calculated value of the diameter, obtained using *TOPOS* (Section 2.3), is 4.3 Å. As shown, the porphyrin units are separated by 9.7187 (4) Å (the *a* cell parameter), allowing the interaction of potential guest molecules with the active metal centres.

**Figure 3:**
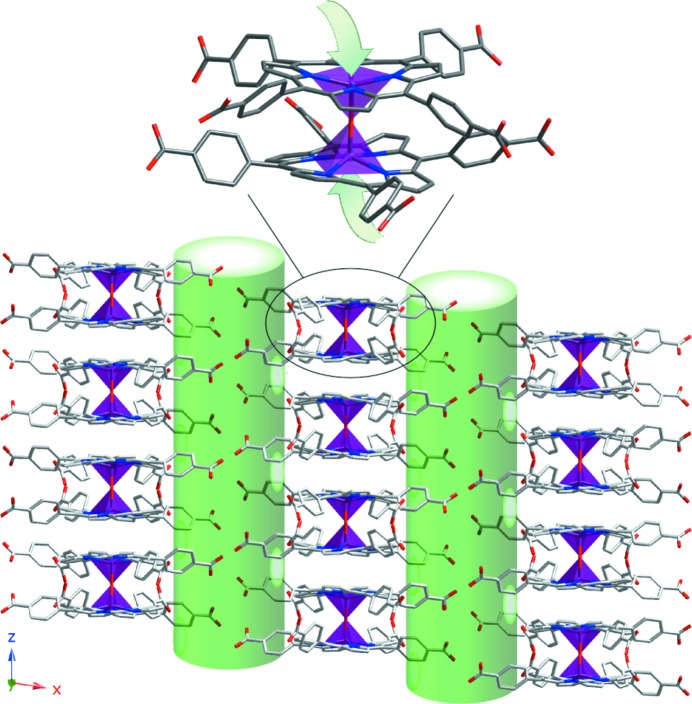
Representation of the accessibility of guest molecules to the active metal centres for compound **2**. Colour code: Fe purple, C grey, N blue and O red. Accessible pathways are represented by the green cylinders.

The crystal structure and thermal analysis of compound **2** (*μ*-*O*-[FeTCPP]_2_·16DMF) have been reported previously by us (Fidalgo-Marijuan *et al.*, 2015 ▸). This compound consists of FeTCPP dimers, where monomers are connected by an O-bridge. In this way, the active catalytic centres are exposed to the channels of the framework. The calculated value of the diameter, obtained using *TOPOS*, is 5.2 Å. As shown in Fig. S6, the dimers are packed forming hydrogen-bonded layers in the *xy* plane, generating the aforementioned channels along the [001] direction, where potential guest molecules are then distributed to the active centres (Fig. 3 ▸).

**Figure 4:**
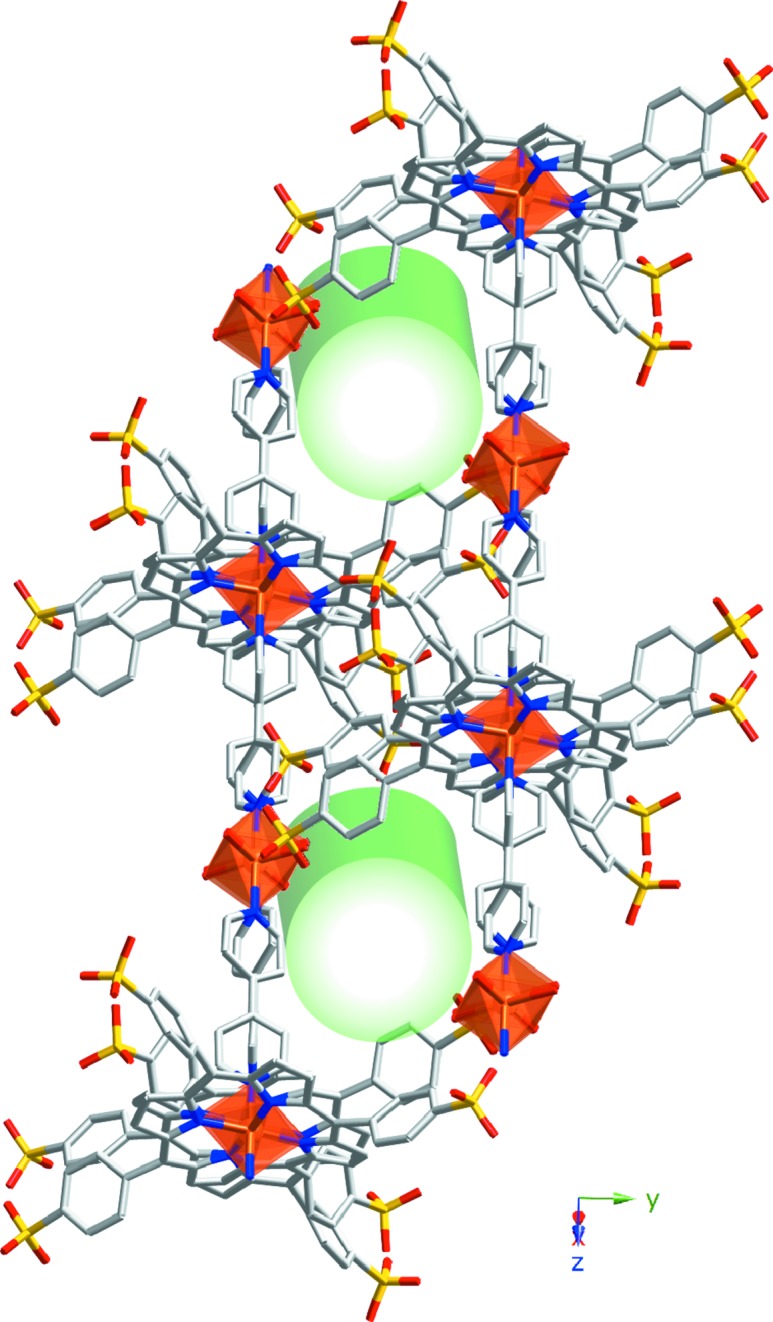
Representation of the accessibility of guest molecules to the active metal centres for compound **3.** Colour code: Co orange, C grey, N blue, O red and S yellow. Accessible pathways are represented by the green cylinders.

The crystal structure and thermal analysis for compound **3**, with the formula [CoTPPS_0.5_(bipy)(H_2_O)_2_]·6H_2_O, have also been reported by us in previous work (Fidalgo-Marijuan *et al.*, 2013 ▸). The crystal structure is formed by one-dimensional polymers, where octahedral Co^II^ ions of CoTPPS units are axially bonded to bipy ligands (Fig. S7). The extension of the one-dimensional polymers consists of a link between alternating Co^II^ centres along the [001] direction through the bipy ligands in a bipy–CoTPPS–bipy–Co(H_2_O)_4_ fashion. In this case, the accessibility to active metal centres takes place along the 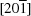 direction where the active metal centres are exposed to the channels of the framework (Fig. 4 ▸). The calculated value for the diameter obtained using *TOPOS* is 6.1 Å.

### 3.2. Thermal analysis   

**Figure 5:**
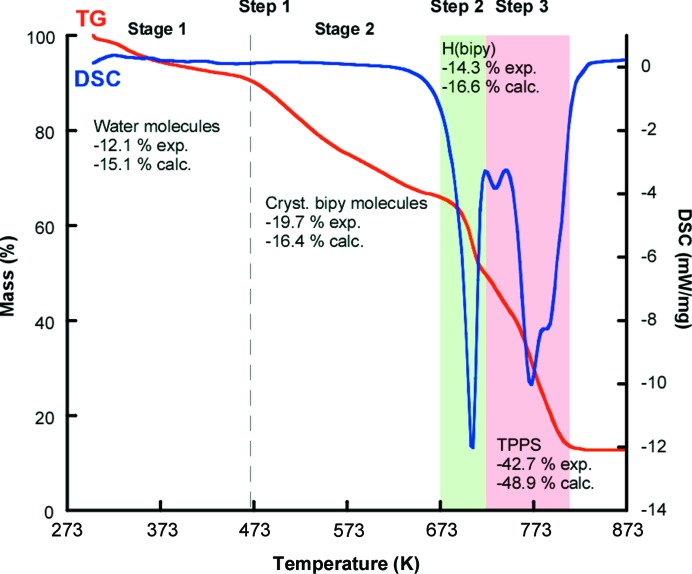
Thermal analysis for compound **1**.

Thermal behaviour is crucial in determining the stability and correct catalytic activation of these materials. As observed in Fig. 5 ▸, the thermogravimetric decomposition curve of compound **1** shows a continuous mass loss from room temperature to 813 K, where three steps can be distinguished. The first is assigned to an overlapped two-stage step between room temperature and 673 K. The first stage occurs up to 468 K, with a 12.1% weight loss attributed to the coordinated and crystallized water molecules. The second stage, up to 673 K (19.7% weight loss), corresponds to the crystallized bi­pyridine molecules. The second step, occurring between 673 K and 723 K, with a 14.3% weight loss, is attributed to the bi­pyridine molecules formerly present as [H(bipy)]^+^ cations. The latter assignment is based on the fact that these cationic entities are more robustly linked than their crystallized analogues. The last step, between 723 K and 813 K (42.7% weight loss), is the final degradation of the TPPS units. The calcination product was identified by X-ray powder diffraction analysis and it consists of Mn_2_O_3_ (space group *R* 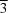 *c*, *a* = 5.04, *c* = 14.12 Å, γ = 120°; Lee *et al.*, 1980 ▸).

Compounds **2** and **3** have been thermally characterized in our previous work (Fidalgo-Marijuan *et al.*, 2015 ▸, 2013 ▸), showing high thermal stability (compound **2** up to 593 K and **3** up to 633 K). The thermogravimetry/differential scanning calorimetry (TG/DSC) curve for activated compound **2** is shown in Fig. S8.

Additionally, Fig. S9 shows the X-ray thermo-diffraction measurements (XRTD) of powdered single crystals for compounds **1** and **2**. The results indicate that both compounds were thermally stable after activation for catalytic purposes.

### 3.3. Catalytic properties   

Synthetic metalloporphyrin complexes have been largely used for a wide variety of catalytic transformations (Chatterjee *et al.*, 2016 ▸; Rayati *et al.*, 2016 ▸; Sengupta *et al.*, 2016 ▸; Zhou *et al.*, 2016 ▸), and in this work we have explored the catalytic activity of the monomeric framework [H(bipy)]_2_[(MnTPPS)(H_2_O)_2_]·2bipy·14H_2_O, **1**, the dimeric framework *μ*-*O*-[FeTCPP]_2_·16DMF, **2** (Fidalgo-Marijuan *et al.*, 2015 ▸), and the one-dimensional framework [CoTPPS_0.5_(bipy)(H_2_O)_2_]·6H_2_O, **3** (Fidalgo-Marijuan *et al.*, 2013 ▸). As previously shown, these three compounds exhibit features that make them suitable candidates for catalysis of different reactions. Firstly, the metal coordination spheres are either unsaturated or there are water molecules that are easy to remove during the activation stage, as shown by the thermogravimetric analysis. Fig. S10 shows a simplified scheme for **1**, **2** and **3**, highlighting the catalytic active centres. In addition, the networks are significantly accessible, with mobile DMF or water solvent molecules located in the cavities.

Thus, the catalytic performance was studied for the oxidation of alcohols, aldol and Knoevenagel condensations, and a one-pot cascade reaction for an acetal hydrolysis followed by a C—C Knoevenagel condensation (Scheme 1). The studied substrates for all the reactions are summarized in Table S5.
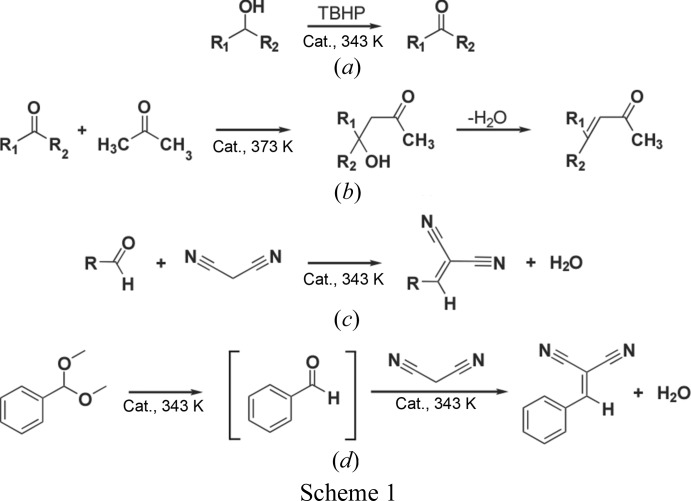


#### 3.3.1. Oxidation of alcohols   

The selective oxidation of alcohols to aldehydes is a relevant transformation in waste recovery and in organic synthesis because of the properties and chemical reactivity of carbonylic compounds that make aldehydes the preferred starting materials in many syntheses (Davis *et al.*, 2013 ▸). Although considerable progress has been made using noble-metal nanoparticles such as Au (Corma & Garcia, 2008 ▸), it would still be desirable to develop catalysts based on less expensive metals and processes. In this sense, some porphyrinic compounds have been tested (Zou *et al.*, 2013 ▸; Machado *et al.*, 2013 ▸; Chen & Ma, 2016 ▸) in an effort to minimize costs and waste generation.

The reaction conditions were initially set using benzyl alcohol as a model substrate. Based on our previous experience (Fidalgo-Marijuan *et al.*, 2015 ▸; Larrea *et al.*, 2011 ▸), the reactions were carried out using *tert*-butyl hydro­peroxide (TBHP) as the oxidizing agent in aceto­nitrile. Using 5% catalyst and 1.5 equivalents of TBHP in 2 ml of solvent at 343 K, total conversions of 50% for **1**, 73% for **2** and 71% for **3** were achieved after 7 h of reaction. The scope of the reaction was studied with various alcohols: 1-phenyl­ethanol, 1-hexanol and 1-octanol (Table 2 ▸). Figs. S11, S12 and S13 show the kinetic profiles of these oxidation reactions for **1**, **2** and **3**.

The best result for **1** is achieved towards the oxidation of 1-hexanol, since **2** is better for 1-phenyl­ethanol. For benzyl alcohol oxidation, the conversion values are quite similar for the three catalysts, though compound **3** clearly shows a higher TOF. The poor conversion values achieved for 1-octanol in all cases could be explained by the fact that this substrate presents more steric hindrance. A comparison of these results with similar porphyrinic catalysts found in the literature indicates a significant reduction in the reaction time (half time) for catalyst **2** (Modak *et al.*, 2013 ▸). Moreover, comparing the benzyl alcohol results with classic Rh-, Ru- and Ce-based catalysts showed the conversion rates are slightly higher and have much shorter reaction times (Wusiman & Lu, 2015 ▸; Burange *et al.*, 2013 ▸; Sarkar *et al.*, 2014 ▸).

**Figure 6:**
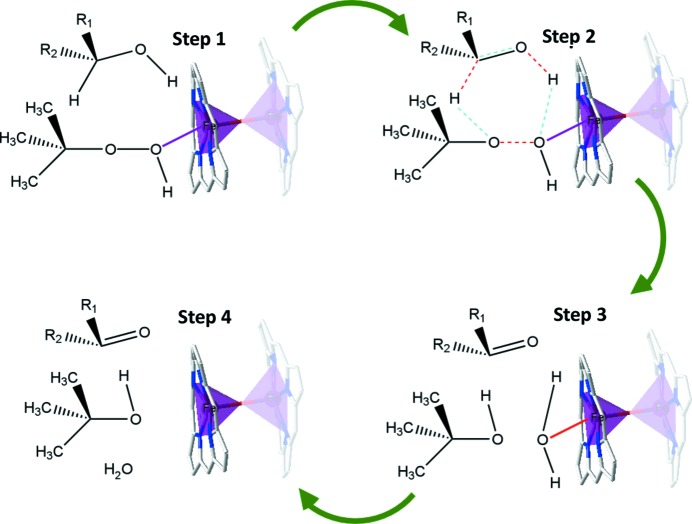
Proposed mechanism for the alcohol oxidations with TBHP over catalyst **2**. Colour code: Fe purple, C grey, N blue and O red. Bonds to be removed and created are marked in red and blue, respectively.

One of the disadvantages of heterogeneous catalysis is the difficulty of studying the reaction mechanisms; often the involved intermediates are unknown. Even so, in the proposed mechanism for alcohol oxidation the initial stage consists of activation of TBHP by coordination to the unsaturated metal centre in order to obtain the corresponding peroxo species. After coordination, *tert*-butoxyl radicals are generated, abstracting an H atom from the substrate and leading to the corresponding aldehyde or ketone (Fig. 6 ▸) (Orive *et al.*, 2013 ▸).

#### 3.3.2. Aldol condensation   

Aldol condensations are important in organic synthesis, providing a way to form C—C bonds; the identification of catalysts capable of performing C—C bond formation remains a challenge (Scheme 1*b* [Chem scheme1]) (Thankachan *et al.*, 2015 ▸). The main drawbacks of using the usual NaOH or KOH catalysts are corrosion problems in the equipment, separation difficulties and the generation of large amounts of waste. To overcome these disadvantages, efforts have been made to design new catalytic systems with controlled basic properties in order to increase the efficiency of the process. In this context, metalloporphyrinic catalysts **1**, **2** and **3** were tested towards the aldol condensation of benz­aldehyde and derivatives with acetone.

The reaction conditions were initially set using benz­aldehyde as the substrate, using 5% catalyst in 1 ml of acetone at 343 K, obtaining poor conversion values (31% for catalyst **1**, 38% for **2** and 2.5% for **3**). However, increasing the catalyst amount to 10% and the reaction temperature to 373 K, the total conversions were 79% for **2** and 16% for **3** after 38 h of reaction. Unfortunately, at this temperature, **1** shows chemical decomposition caused by the loss of the [H(bipy)]^+^ cations. Thus, the scope of the reaction was studied with *p*-tolu­aldehyde, *p*-methoxybenzaldehyde and heptanal using compound **2** as the catalyst. Table 3 ▸ and Fig. S14 show the conversion values and kinetic profiles for the aldol condensation, respectively.

As observed, the methyl group in the *para* position leads to high yields, whereas meth­oxy groups give poor conversion. In the case of heptanal, the reaction evolves much more quickly, giving rise to the heptanal autocondensation product (Fig. S15*a*) in a minority amount (7%) and two isomers (double-bond position) of the aldol condensation: the dec-3-en-2-one (52%) (Fig. S15*b*) and the dec-4-en-2-one (35%) (Fig. S15*c*). Comparing the results with other MOF-type catalysts, we conclude that, for aromatic substrates, it is necessary to increase the reaction time considerably (up to three times) to obtain similar conversion rates (Abedi *et al.*, 2016 ▸). Nevertheless, the reaction with heptanal shows excellent activity using four times less catalyst than in similar reported studies (Yadav & Aduri, 2012 ▸).

#### 3.3.3. Knoevenagel condensation   

Taking into account the previous results, compound **2** was also tested as a catalyst for the Knoevenagel condensation reaction (Scheme 1*c* [Chem scheme1]) between various substrates and derivatives (Table 4 ▸) and malono­nitrile (p*K*_a_ = 11.1). As above, the reaction conditions were set using benzaldehyde as the substrate, 5% catalyst, 1.0 equivalent of malono­nitrile in 2 ml of toluene and 2 µl of do­decane as internal standard at 343 K, reaching a total conversion of 79% after 22 h of reaction (Table 4 ▸). The scope of the reaction was then studied with *p*-tolu­aldehyde, *p*-fluoro­benzaldehyde, 4-chloro­benzaldehyde and 3-nitro­benzaldehyde. Fig. S16 shows the kinetic profiles of the Knoevenagel condensation reactions.

As observed, when introducing substituents into the *meta*- or *para*-positions of the benzaldehyde ring, the conversion rates differ as a result of electronic effects, following the order of reactivity: *m*-nitro­benzaldehyde ≃ *p*-chloro­benz­aldehyde > *p*-tolu­aldehyde ≃ benzaldehyde > *p*-fluoro­benz­aldehyde.

**Figure 7:**
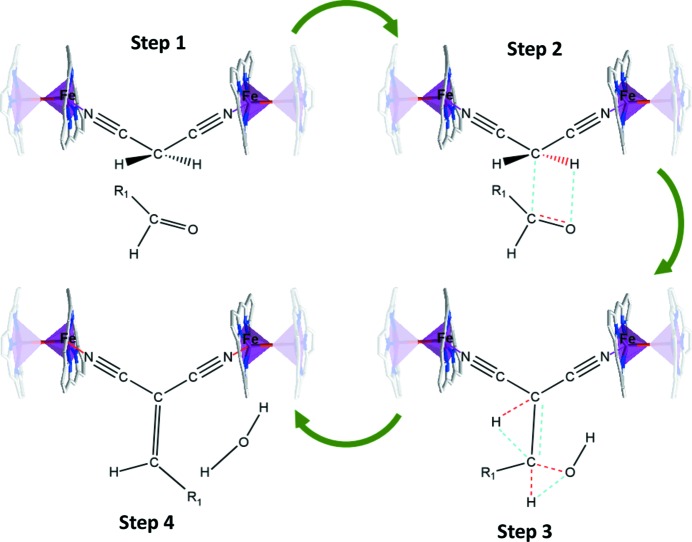
Proposed mechanism for the benzyl alcohol Knoevenagel condensation over catalyst **2**. Colour code: Fe purple, C grey, N blue and O red. Bonds to be removed (red dashed lines) and created (turquoise dashed lines) are shown.

For both aldol and Knoevenagel condensations, the activation of the carbonyl groups is prompted by a Lewis acid (the metal ion). The proximity of the active centres (Fe ions) meant that a mechanism involving two-site adsorption of the donor molecule could be proposed (Fig. 7 ▸) (Larrea *et al.*, 2015 ▸; Položij *et al.*, 2014 ▸). In fact, the distance of 5.656 (1) Å between Fe^III^ ions allowed the cyanide groups of malono­nitrile [N⋯N distance 4.319 (3) Å] to interact with the Fe active centres. Comparing these results with other MOFs (Larrea *et al.*, 2015 ▸), the obtained conversion rates and TOF are among the best using only 5% catalyst under similar reaction conditions.

#### 3.3.4. One-pot cascade reaction   

Taking into account that compound **2** showed the best results for Knoevenagel condensation, it was tested in a bifunctional one-pot cascade reaction (Scheme 1*d* [Chem scheme1]). The first step involves an acetal hydrolysis using benzaldehyde di­methyl acetal as the substrate, followed by a second step in which Knoevenagel condensation with malono­nitrile takes place. The results (TOF: 10 h^−1^, CT: 42% in 44 h) indicate that, although the Knoevenagel condensation reaches a high conversion (79%; Table 4 ▸), in this case the rate-limiting step is the reaction involving the acetal hydrolysis. Although in the first few hours the TOF is quite high, after 3 h the conversion rate decelerates, probably because of the inability of the catalyst to overcome the first step of the reaction (Fig. S17).

An excellent strategy to carry out this type of one-pot two-step reaction is to introduce, in the same solid catalyst, both acidic and basic active centres (Merino *et al.*, 2013 ▸). Compound **2** shows only Lewis acid centres to catalyse the hydrolysis of benzaldehyde di­methyl­acetal into benzaldehyde and react with malono­nitrile through Knoevenagel condensation. This may be the reason for the lower conversion in the one-pot two-step reaction (42%) compared with the single Knoevenagel condensation (79%) for **2**.

### 3.4. Heterogeneity and recyclability tests   

**Figure 8:**
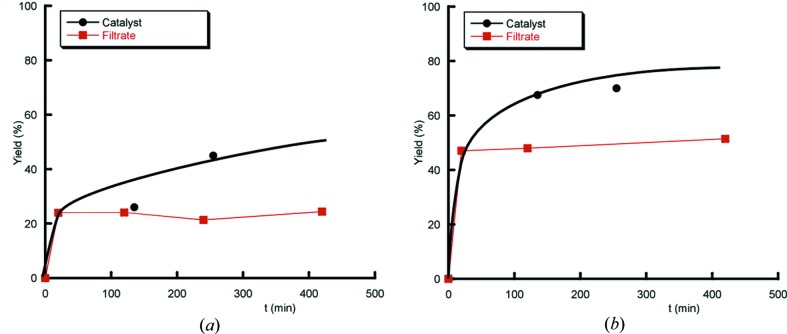
Kinetic profiles of the oxidation of benzyl alcohol (*a*) over compound **1** and (*b*) over compound **3** after hot filtering with TBHP.

The heterogeneous nature of catalysts **1** and **3** towards the oxidation of alcohols were tested using benzyl alcohol (**2** has been tested previously; Fidalgo-Marijuan *et al.*, 2015 ▸). For rigorous proof of heterogeneity, a test (Sheldon *et al.*, 1998 ▸) was carried out by filtering the catalyst from the reaction mixture at 343 K after 20 min, when conversions of 24% and 47% had been reached for **1** and **3**, respectively. The filtrate was allowed to react for up to 7 h. The reaction mixture and the filtrate were analysed afterwards by GC–MS. No significant change in the conversion rate was found for the filtrate (Fig. 8 ▸), meaning that the active species does not leach and the observed catalysis is truly heterogeneous in nature.

The recyclability of catalysts **1** and **3** was also tested for the benzyl alcohol oxidation. The catalyst was recovered after the reaction by centrifugation and washed several times with aceto­nitrile, then dried at 373 K and reused. As shown in Table S6, catalyst **3** maintains activity after five cycles, whereas **1** shows a small decrease.

**Figure 9:**
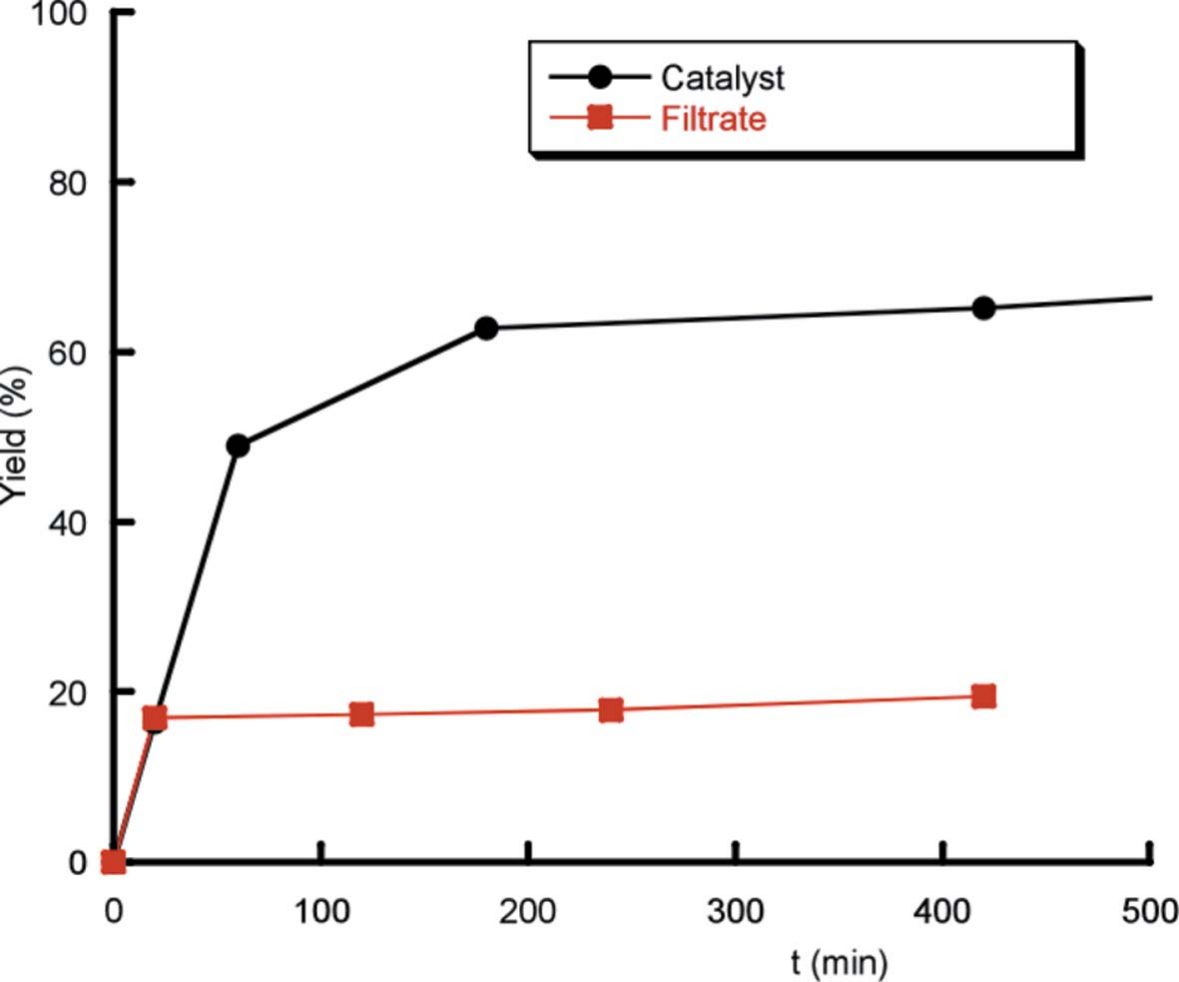
Kinetic profile of the condensation of benzaldehyde with malono­nitrile over compound **2** after hot filtering.

As was previously done for the oxidation reactions, the heterogeneous nature of the catalyst and recyclability were tested or the aldol and Knoevenagel condensations by hot filtration. As shown in Fig. 9 ▸, the experiments reveal that **2** is a truly heterogeneous catalyst for this reaction. Additionally, the catalyst was reused over five cycles and shows a progressive decrease in catalytic activity after the third cycle (Table S7).

After the catalytic reactions, the catalysts were recovered by centrifugation, washed with aceto­nitrile, ethanol or toluene and then characterized by IR spectroscopy. The IR spectra of the recovered catalyst for all reactions showed that the chemical-bond systems remained unchanged (Fig. S18). In fact, the solid shows the same characteristic vibration modes as the original compound. As shown in Fig. S18, the characteristic vibrations of the porphyrin macrocycle are present. Additionally, both the fresh catalyst and the recovered solid after the reaction were studied by TEM, as discussed below.

### 3.5. Transmision electron microscopy   

In order to carry out a deeper characterization of the recovered catalyst, compounds **2** and **3** were analysed by TEM before and after the catalytic reactions. Compound **1** is unstable under an electron beam, so it was not analysed by TEM. TEM analysis shows that compounds **2** and **3** maintain a certain grade of crystallinity following the catalytic reactions. Moreover, the pre- and post-catalysis particles of **2** and **3** keep their morphology: elongated prisms for **2** and curved-edge particles for **3** (Fig. S19).

**Figure 10:**
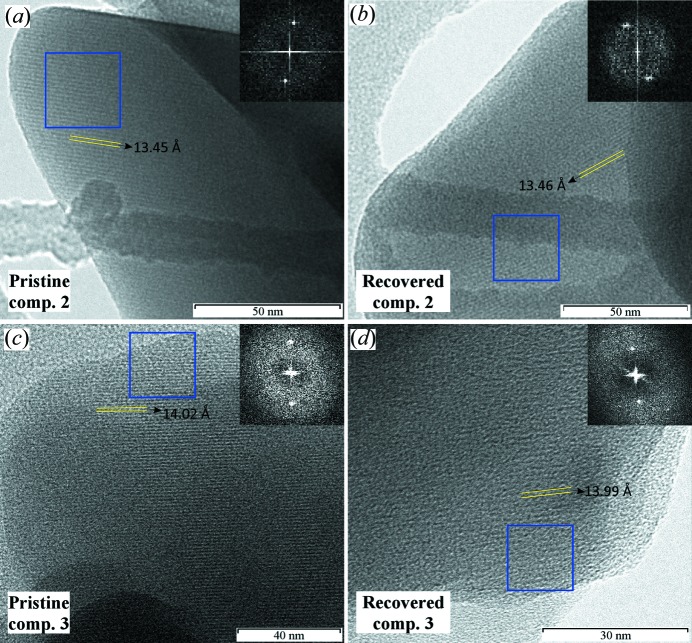
HRTEM images for pristine compounds (*a*) **2** and (*c*) **3** and the recovered residues after the catalytic reactions for (*b*) **2** and (*d*) **3**. Lattice spacing is marked by yellow lines and the upper right image corresponds to the Fourier transform of the area inside the blue square.

The crystalline nature and moderate stability of samples **2** and **3** under an electron beam allowed us to measure the lattice spacing for both compounds. The HRTEM images (Fig. 10 ▸) of the pristine sample (*a*) and the recovered residue after the catalytic reaction (*b*) of compound **2** reveal a lattice spacing of 13.45 Å along the width of the crystal. This observed lattice spacing corresponds to the (101), 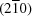 and (210) set of crystallographic planes. For compound **3**, the pristine sample (*c*) and the recovered residue (*d*) present a spacing near to 14.02 Å, which corresponds to the (011) set of crystallographic planes. Therefore, both compounds maintain the same lattice spacing before and after the catalytic reactions so, as previously observed by IR spectroscopy, compounds **2** and **3** keep their structural integrity.

## 4. Conclusions   

This work explores an innovative approach that consists of using metalloporphyrins as both synthons and catalytic units in solid coordination networks. This strategy is compatible with green synthesis, *i.e.* using first-row transition metals (Mn, Fe and Co), with water as the preferred solvent and performing syntheses at low temperatures. While most of the studies involving solid coordination networks are focused on three-dimensional covalent MOFs, the results presented here indicate that accessibility of reactants to the metal centres is a significant parameter in achieving good heterogeneous catalytic activity. The studied compounds are perfectly operative under ambient conditions as they exhibit recyclability and thermochemical stability. Furthermore, compounds **2** and **3** are remarkably stable upon heating. An improvement in catalytic performance has been achieved in relation to commonly used toxic metal-based catalysts. In summary, bioinspired metalloporphyrinic SMOFs are promising candidates as heterogeneous and recyclable catalysts.

## Supplementary Material

Crystal structure: contains datablock(s) global, I. DOI: 10.1107/S2052252518007856/lq5011sup1.cif

Supporting figures and tables. DOI: 10.1107/S2052252518007856/lq5011sup2.pdf

CCDC reference: 1515741

## Figures and Tables

**Table 1:** Crystallographic data for **1**

Compound	[H(bipy)]_2_[(MnTPPS)(H_2_O)_2_]·2bipy·14H_2_O	
Formula	C_42_H_46_Mn_0.5_N_6_O_14_S_2_	
FW (g mol^−1^)	950.44	
Crystal system	Triclinic	
Space group (No. 2)	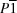	
*a* (Å)	9.7187 (4)	
*b* (Å)	11.2496 (5)	
*c* (Å)	21.8708 (7)	
*α* (°)	88.401 (3)	
*β* (°)	83.848 (3)	
*γ* (°)	64.446 (4)	
*V* (Å^3^)	2144.4 (2)	
*Z*	2	
ρ_obs_, ρ_cal_ (g cm^−3^)	1.44 (5), 1.472	
*F*(000)	993	
*μ* (mm^−1^)	2.923	
Crystal size (mm)	0.14 × 0.05 × 0.01	
Absorption correction	Analytical	
Radiation, λ (Å)	1.54184	
Temperature (K)	100.0 (2)	
Reflections collected, unique	17468, 8113 (*R*_int_ = 0.051)	
Limiting indices	−9 ≤*h* ≤ 11, −13 ≤ *k* ≤ 13, −26 ≤ *l* ≤ 26	
Refinement method	Full-matrix least-squares on *F* ^2^	
Final *R* indices [I > 2σ(*I*)]†	*R*1 = 0.0609, w*R*2 = 0.1516	
*R* indices (all data)†	*R*1 = 0.0984, w*R*2 = 0.1742	
Goodness of fit on *F* ^2^	1.012	
Parameters, restraints	599, 4	

† *R*1 = [(|*F*_o_|−|*F*_c_|)]/|*F*_o_|. w*R*2 = [[w|*F*_o_|^2^−|*F*_c_|^2^)^2^]/[*w*(|*F*_o_|^2^)^2^]^1/2^.

**Table 2:** Selective oxidation of several alcohols over catalysts **1, 2** and **3** C_T_ is the total conversion.

		Compound **1**			Compound **2**			Compound **3**		
Substrate	Oxidant	TOF (h^−1^)	C_T_ (%)	t (h)	TOF (h^−1^)	C_T_ (%)	t (h)	TOF (h^−1^)	C_T_ (%)	t (h)
Benzyl-alcohol	TBHP	72	70	20	72	73	7	143	77	20
1-Phenyl­ethanol	TBHP	46	44	24	91	73	5	8	44	21
1-Hexanol	TBHP	66	92	24	3	15	6	22	71	24
1-Octanol	TBHP	2	12	24	6	9	18	2	25	24

**Table 3:** Aldol condensation with acetone over catalyst **2**

Substrate	TOF (h^−1^)	C_T_ (%)	t (h)
Benzaldehyde	3.5	79	93
*p*-Tolu­aldehyde	2.3	92	87
*p*-Methoxybenzaldehyde	1.4	50	87
Heptanal	152	94	4

**Table 4:** Knoevenagel condensation over catalyst **2**

Substrate	TOF (h^−1^)	C_T_ (%)	t (h)
Benzaldehyde	49	79	22
*p*-Tolu­aldehyde	53	81	24
*p*-Fluoro­benzaldehyde	32	69	24
*p*-Chloro­benzaldehyde	137	100	24
*m*-Nitro­benzaldehyde	129	100	18
