# Supplementary material for: Double role of metalloporphyrins in catalytic bioinspired supramolecular metal–organic frameworks (SMOFs)
Source: IUCrJ. 2018 Jul 20;5(Pt 5):559–68. doi: 10.1107/S2052252518007856 (PMC6126652; doi:10.1107/S2052252518007856)
Supplement: Supplementary file 2 [file m-05-00559-sup2.pdf]

IUCrJ

Volume 5 (2018)

Supporting information for article:

Double role of metalloporphyrins in catalytic bioinspired supramolecular metal–organic frameworks (SMOFs)

Arkaitz Fidalgo-Marijuan, Eder Amayuelas, Gotzone Barandika, Edurne S. Larrea, Begoña Bazán, Miren Karmele Urtiaga, Marta Iglesias and Maribel Arriortua

Double role of metalloporphyrins in catalytic bioinspired supramolecular Metal-Organic Frameworks (SMOFs)

Arkaitz Fidalgo-Marijuan[ab], Eder Amayuelas[a], Gotzone Barandika[cb]*, Edurne S. Larrea[a], Begoña Bazán[ab], Miren Karmele Urtiaga[a], Marta Iglesias[d] and Maribel Arriortua[ab]

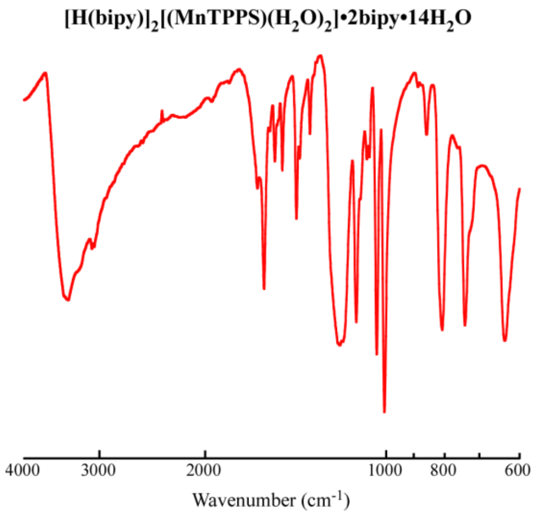

Figure S1 IR spectra for compound **1**.

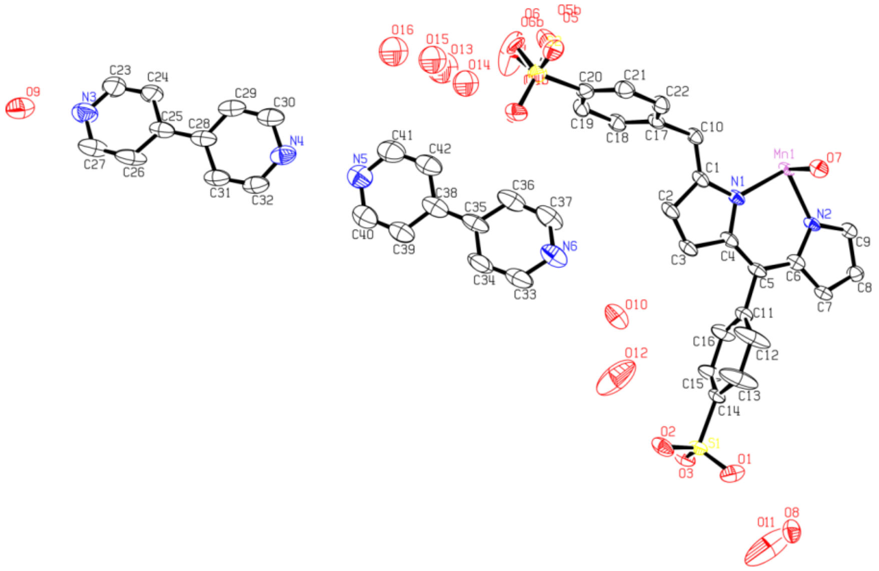

Figure S2 ORTEP detail for compound **1**. The thermal ellipsoids correspond to 50% probability. Color code: Mn=Purple, C=black, N=blue, O=red, S=yellow. Hydrogen atoms have been omitted.

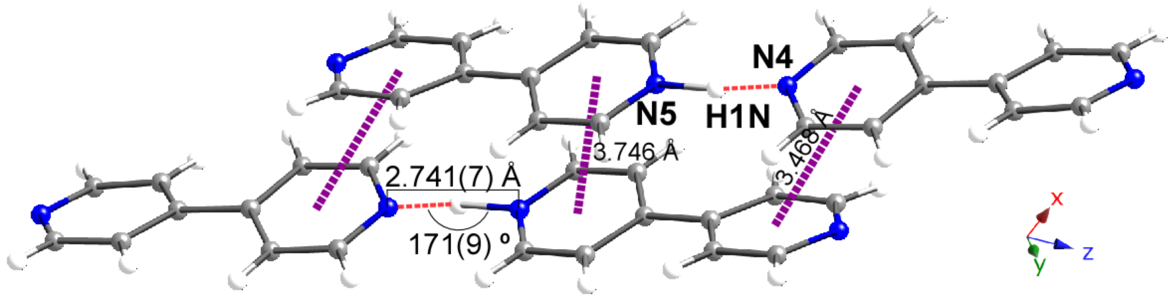

Figure S3 Detail of the $[H(bipy)]^+$ cations for $[H(bipy)]_2[(MnTPPS)(H_2O)_2]·2bipy·14H_2O$ **(1)**. Hydrogen bonds are marked in red dashed lines and π-π interactions in purple dashed lines. Color code: C, grey; N, blue; and H, white.

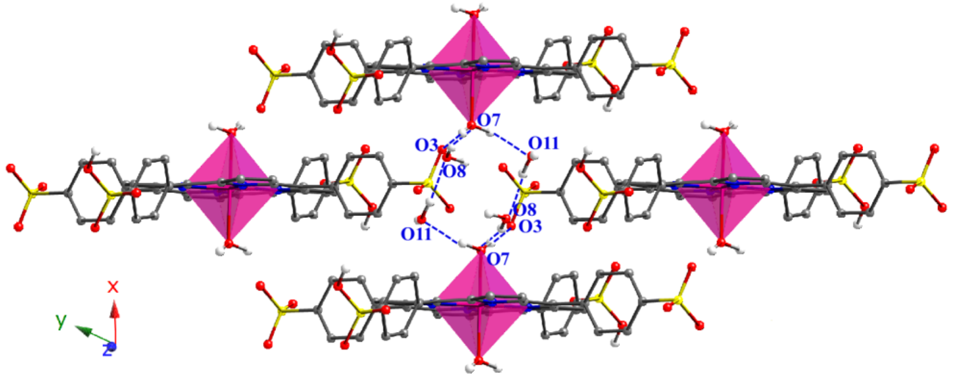

Figure S4 Detail for the H-bonding system in compound **1**. Color code: Mn, pink; C, grey; N, blue; O, red; S, yellow and H, white. Hydrogen bonds are marked as blue dashed lines. Porphyrin ring H atoms and H(bipy) cations have been omitted for clarity.

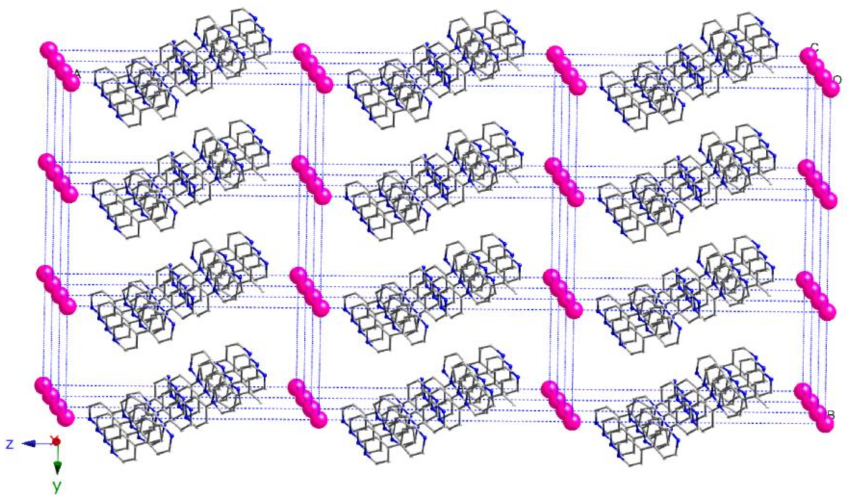

Figure S5 Simplification of the structure for compound **1** showing the relative position of the $[H(bipy)]^+$ cations inside the Mn-node net. Color code: Mn, pink; C, grey and N, blue.

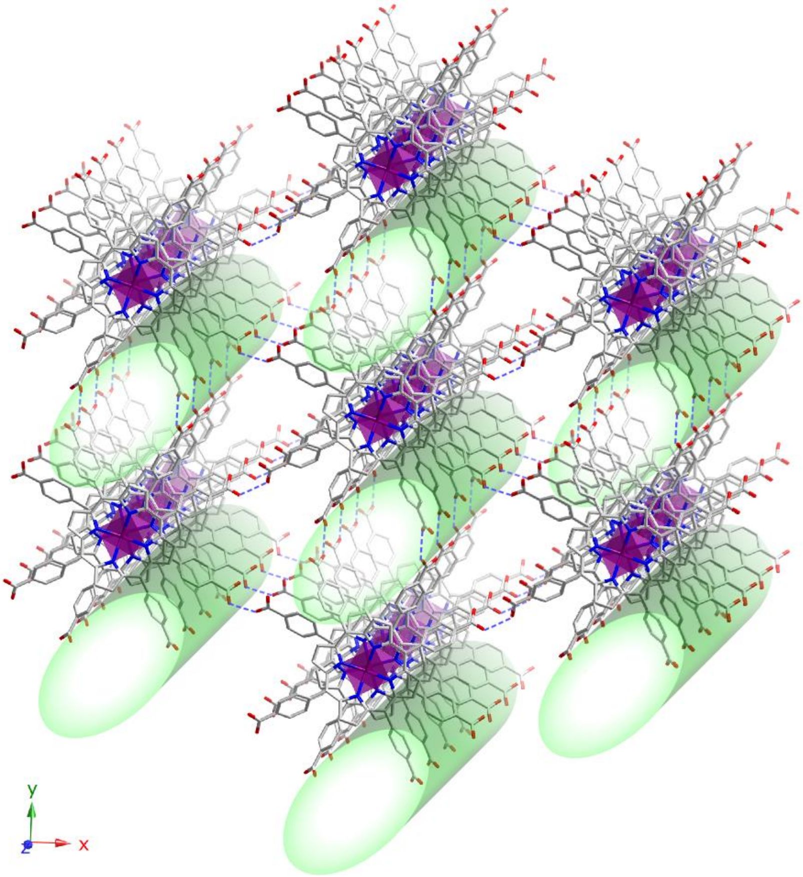

Figure S6 Perspective view of the stacked *xy* H-bonded layers for compound μ-O-[FeTCPP]$_2$·16DMF **(2)**. Accessible pathways are represented with green cylinders. Color code: Fe, purple; C, grey; N, blue and O, red.

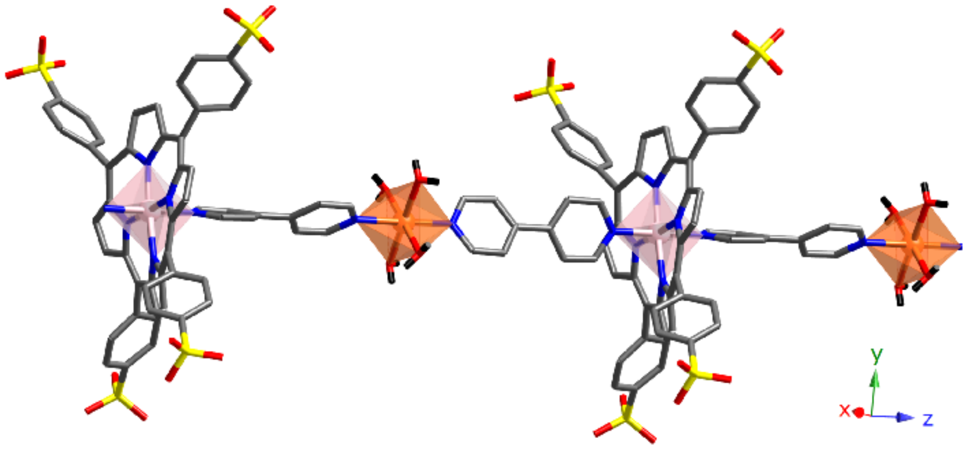

Figure S7 Detail of the structure for compound [CoTPPS$_{0.5}$(bipy)(H$_2$O)$_2$]·6H$_2$O **(3)** showing the extension of the 1D polymers. Color codes: Co(1) (TPPS) in pink, Co(2) in orange, N in blue, C in grey, O in red, S in yellow. H atoms (except those of water molecules, in black) have been omitted for clarity.

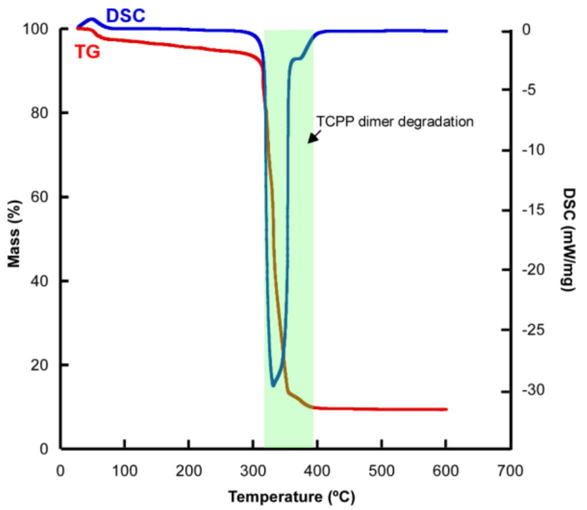

Figure S8 Thermogravimetric analysis (TG/DSC) for activated compound **2**.

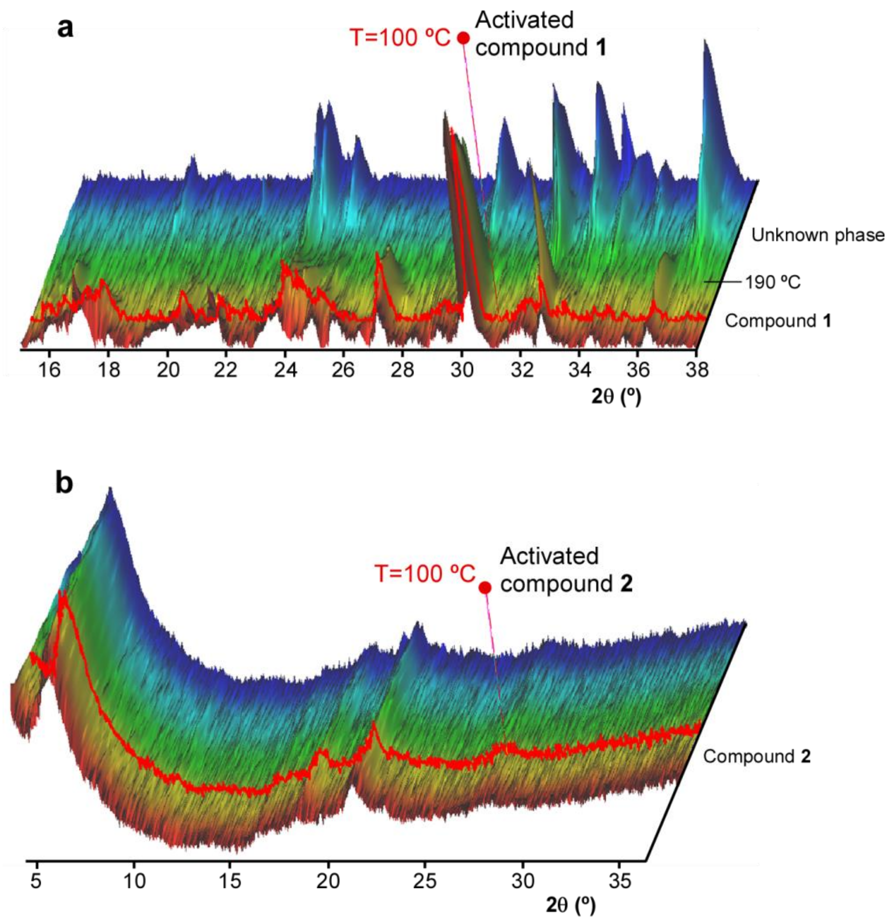

Figure S9 X-ray thermo-diffraction analysis for compounds **1** (a) and **2** (b).

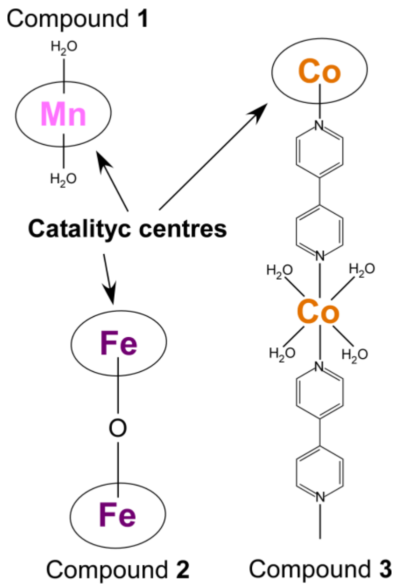

Figure S10 Simplified scheme for compounds **1**, **2** and **3**. The porphyrin ring is represented with a black circle (TPPS for **1** and **3**, and TCPP for **2**).

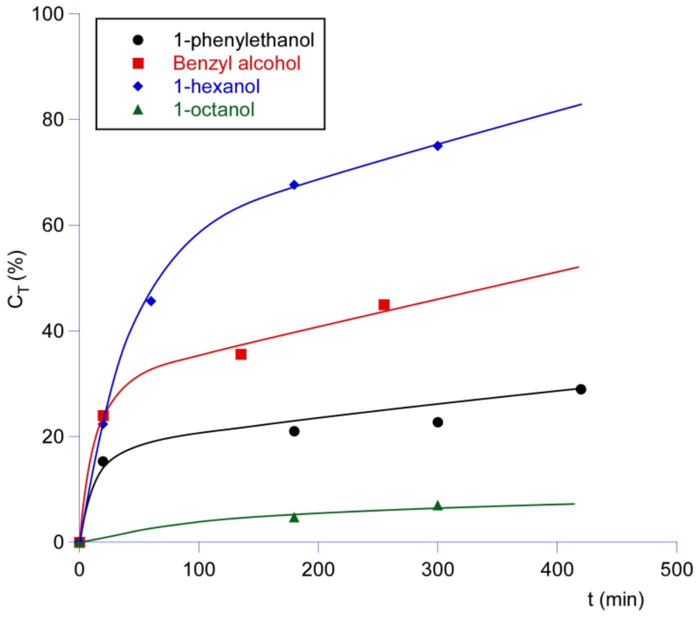

Figure S11 Kinetic profiles for alcohols oxidation using compound **1** as catalyst.

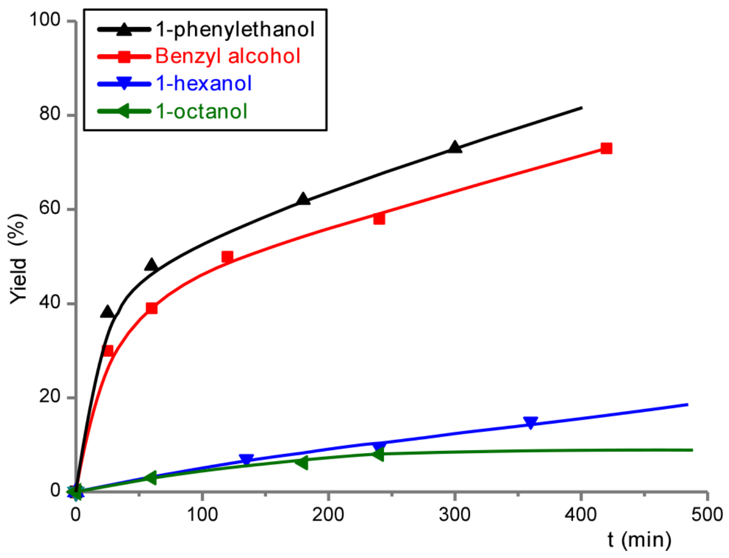

Figure S12 Kinetic profiles for alcohols oxidation using compound **2** as catalyst.

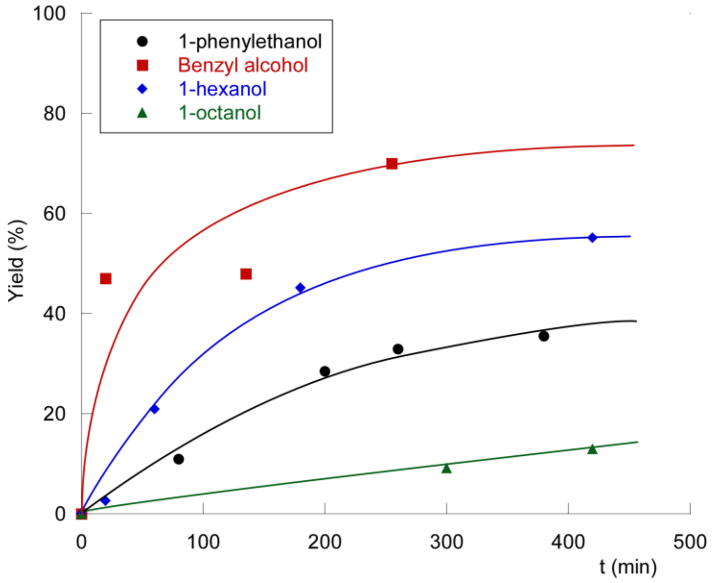

Figure S13 Kinetic profiles for alcohols oxidation using compound **3** as catalyst.

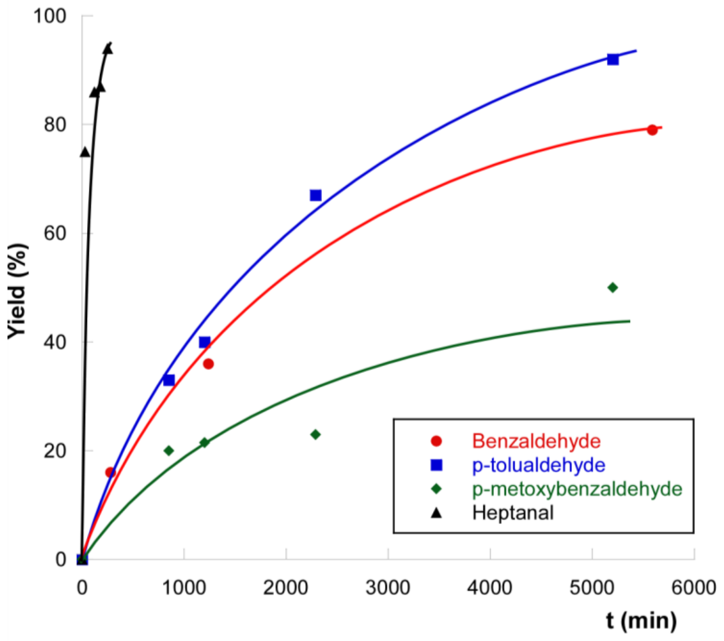

Figure S14 Kinetic profiles for the aldol condensation using compound **2** as catalyst.

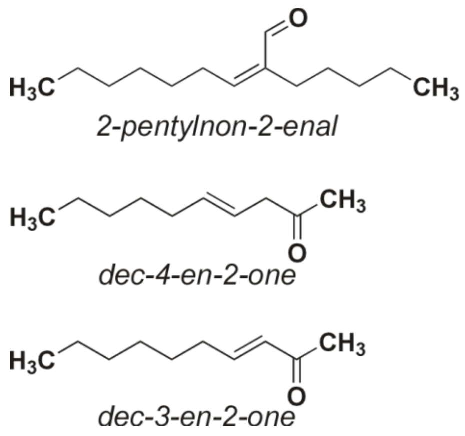

a

b

c

Figure S15 Aldol condensation products for the heptanal substrate.

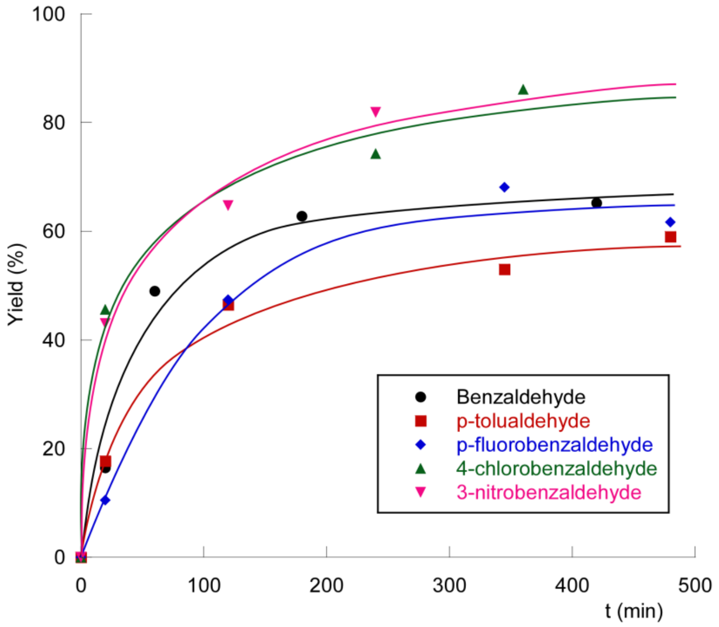

Figure S16 Kinetic profile for the Knoevenagel condensation using compound **2** as catalyst.

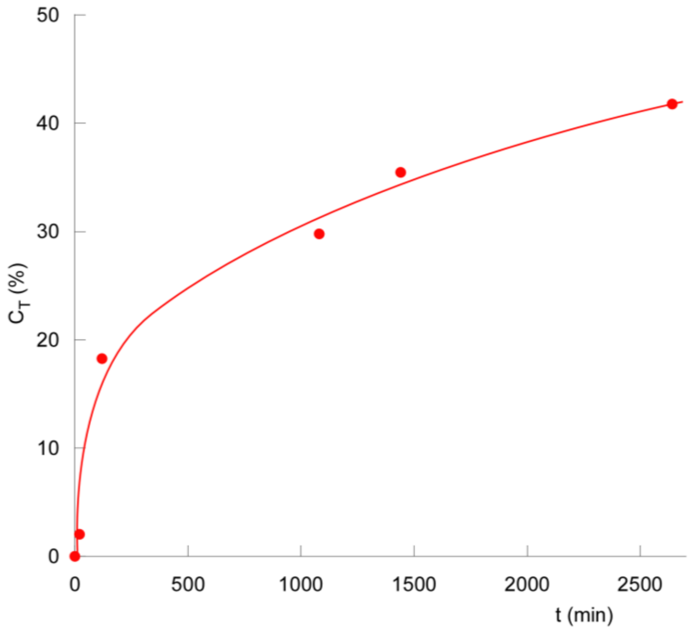

Figure S17 Kinetic profile for the acetal hydrolysis and Knoevenagel condensation one-pot cascade reaction using compound **2** as catalyst.

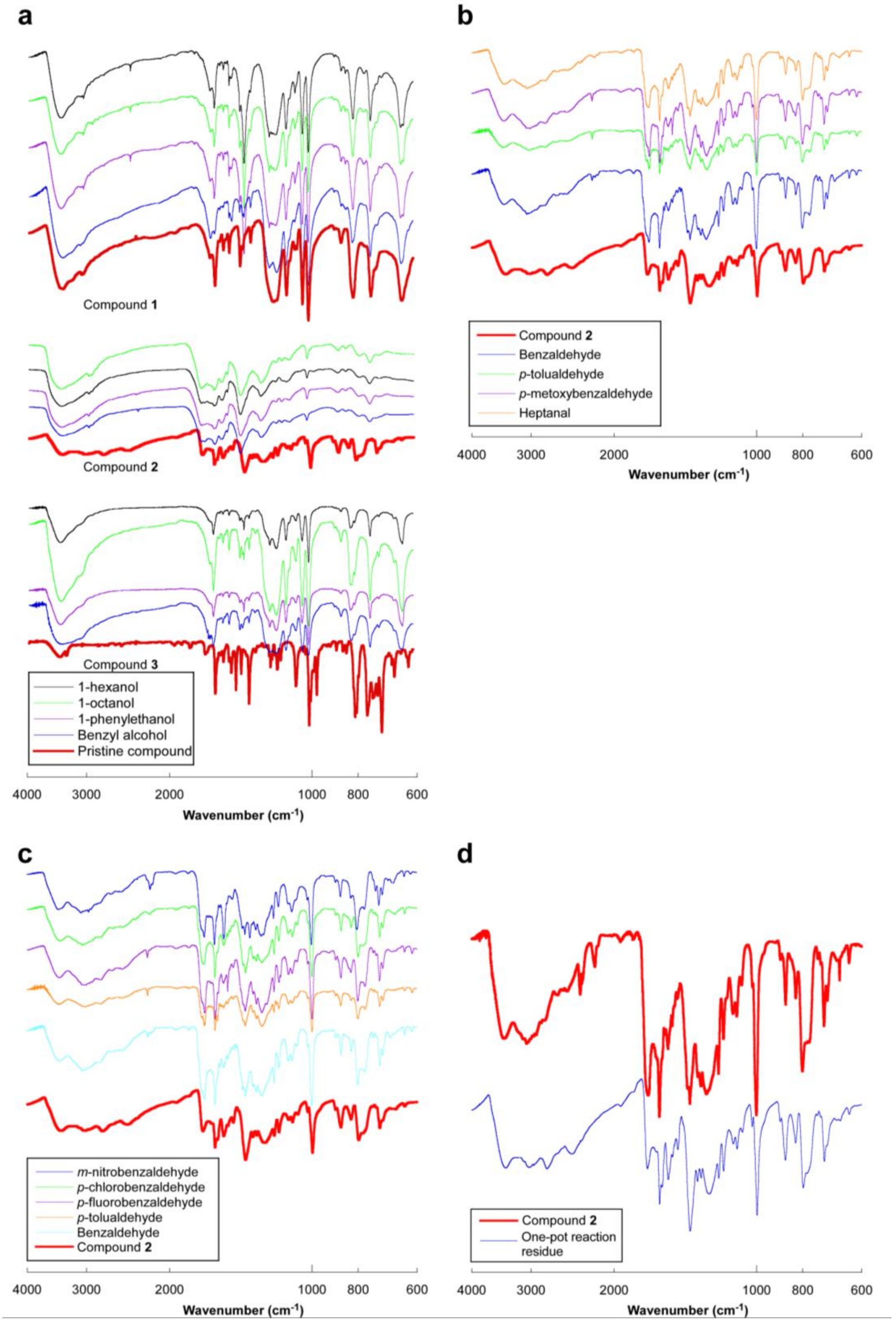

Figure S18 Infrared spectra for the recovered residues after oxidation reactions (a), aldol condensations (b), Knoevenagel condensations (c) and acetal hydrolysis and Knoevenagel condensation one-pot cascade reaction (d).

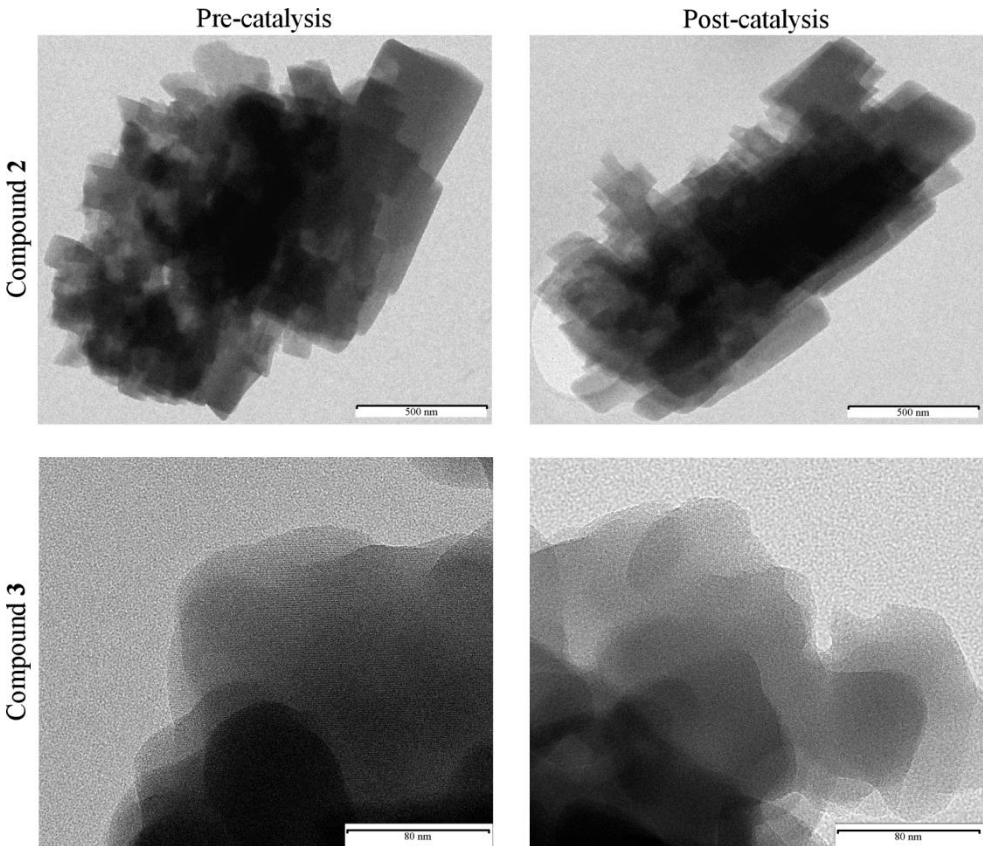

Figure S19 TEM particles morphology for compound **2** and **3** before and after the catalytic reactions.

Table S1 Bond distances (Å) and angles (º) for compound **1**.

Distances

C1—N1	1.376 (5)	C33—C34	1.374 (9)
C1—C10	1.397 (5)	C33—H33	0.95
C1—C2	1.437 (5)	C34—C35	1.377 (8)
C2—C3	1.350 (6)	C34—H34	0.95
C2—H2	0.95	C35—C36	1.397 (7)
C3—C4	1.436 (5)	C35—C38	1.462 (8)
C3—H3	0.95	C36—C37	1.374 (9)
C4—N1	1.380 (5)	C36—H36	0.95
C4—C5	1.390 (5)	C37—N6	1.328 (7)
C5—C6	1.392 (5)	C37—H37	0.95
C5—C11	1.500 (5)	C38—C39	1.393 (7)
C6—N2	1.383 (5)	C38—C42	1.405 (8)
C6—C7	1.427 (5)	C39—C40	1.357 (8)
C7—C8	1.345 (5)	C39—H39	0.95
C7—H7	0.95	C40—N5	1.346 (7)
C8—C9	1.437 (5)	C40—H40	0.95
C8—H8	0.95	C41—N5	1.338 (8)
C9—N2	1.381 (5)	C41—C42	1.361 (9)
C9—C10^{i}	1.393 (5)	C41—H41	0.95
C10—C9^{i}	1.393 (5)	C42—H42	0.95
C10—C17	1.494 (5)	N1—Mn1	2.012 (3)
C11—C12	1.365 (6)	N2—Mn1	2.011 (3)
C11—C16	1.377 (6)	N5—H1N	1.28 (9)
C12—C13	1.381 (7)	O1—S1	1.446 (3)
C12—H12	0.95	O2—S1	1.451 (3)
C13—C14	1.377 (6)	O3—S1	1.458 (3)
C13—H13	0.95	O4—S2	1.42 (4)
C14—C15	1.356 (6)	O5—S2	1.465 (16)
C14—S1	1.776 (3)	O6—S2	1.425 (15)
C15—C16	1.384 (6)	O6—H6	0.851 (10)
C15—H15	0.95	O6—H6B	1.13 (5)
C16—H16	0.95	O4B—S2	1.46 (4)
C17—C22	1.388 (5)	O5B—S2	1.44 (2)
C17—C18	1.391 (6)	O6B—S2	1.41 (2)
C18—C19	1.390 (6)	O6B—H6	0.62 (4)
C18—H18	0.95	O6B—H6B	0.851 (10)
C19—C20	1.379 (6)	O7—Mn1	2.245 (3)

C19—H19	0.95	O7—H7A	0.8208
C20—C21	1.384 (6)	O7—H7B	0.8182
C20—S2	1.785 (4)	Mn1—N2^{i}	2.011 (3)
C21—C22	1.391 (5)	Mn1—N1^{i}	2.012 (3)
C21—H21	0.95	Mn1—O7^{i}	2.245 (3)
C22—H22	0.95	O8—H8A	0.8226
C23—N3	1.321 (8)	O8—H8B	0.8258
C23—C24	1.373 (11)	O9—H9A	0.8296
C23—H23	0.95	O9—H9B	0.8297
C24—C25	1.382 (8)	O10—H10A	0.8263
C24—H24	0.95	O10—H10B	0.8274
C25—C26	1.390 (8)	O11—H11A	0.8201
C25—C28	1.476 (8)	O11—H11B	0.8214
C26—C27	1.356 (9)	O12—H12B	0.8219
C26—H26	0.95	O12—H12A	0.8233
C27—N3	1.318 (7)	O13—H13B	0.8213
C27—H27	0.95	O13—H13A	0.8256
C28—C29	1.394 (7)	O13—H14A	1.2319
C28—C31	1.400 (6)	O13—H16B	1.441
C29—C30	1.361 (8)	O15—H15A	0.8237
C29—H29	0.95	O15—H15B	0.8244
C30—N4	1.345 (7)	O14—H13A	1.0074
C30—H30	0.95	O14—H14A	0.8156
C31—C32	1.350 (8)	O14—H14B	0.8249
C31—H31	0.95	O16—H13B	1.2329
C32—N4	1.342 (7)	O16—H16A	0.8327
C32—H32	0.95	O16—H16B	0.8243
C33—N6	1.342 (7)		

**Angles**		N6—C33—H33	118.3
N1—C1—C10	126.0 (3)	C34—C33—H33	118.3
N1—C1—C2	109.6 (3)	C33—C34—C35	120.7 (5)
C10—C1—C2	124.3 (3)	C33—C34—H34	119.6
C3—C2—C1	107.2 (3)	C35—C34—H34	119.6
C3—C2—H2	126.4	C34—C35—C36	115.5 (6)
C1—C2—H2	126.4	C34—C35—C38	122.2 (5)
C2—C3—C4	107.5 (3)	C36—C35—C38	122.3 (5)
C2—C3—H3	126.3	C37—C36—C35	120.6 (5)
C4—C3—H3	126.3	C37—C36—H36	119.7
N1—C4—C5	126.3 (3)	C35—C36—H36	119.7

N1—C4—C3	109.4 (3)	N6—C37—C36	123.4 (5)
C5—C4—C3	124.4 (3)	N6—C37—H37	118.3
C4—C5—C6	124.6 (3)	C36—C37—H37	118.3
C4—C5—C11	117.6 (3)	C39—C38—C42	115.8 (6)
C6—C5—C11	117.9 (3)	C39—C38—C35	121.5 (5)
N2—C6—C5	125.4 (3)	C42—C38—C35	122.7 (5)
N2—C6—C7	109.7 (3)	C40—C39—C38	120.9 (5)
C5—C6—C7	124.8 (3)	C40—C39—H39	119.5
C8—C7—C6	107.5 (3)	C38—C39—H39	119.5
C8—C7—H7	126.3	N5—C40—C39	121.9 (6)
C6—C7—H7	126.3	N5—C40—H40	119
C7—C8—C9	107.6 (3)	C39—C40—H40	119
C7—C8—H8	126.2	N5—C41—C42	121.8 (6)
C9—C8—H8	126.2	N5—C41—H41	119.1
N2—C9—C10^{i}	126.4 (3)	C42—C41—H41	119.1
N2—C9—C8	109.2 (3)	C41—C42—C38	120.8 (6)
C10^{i}—C9—C8	124.4 (3)	C41—C42—H42	119.6
C9^{i}—C10—C1	123.2 (3)	C38—C42—H42	119.6
C9^{i}—C10—C17	117.8 (3)	C1—N1—C4	106.3 (3)
C1—C10—C17	119.0 (3)	C1—N1—Mn1	127.4 (2)
C12—C11—C16	118.4 (4)	C4—N1—Mn1	126.3 (2)
C12—C11—C5	121.4 (4)	C9—N2—C6	106.0 (3)
C16—C11—C5	120.2 (4)	C9—N2—Mn1	127.1 (2)
C11—C12—C13	121.1 (4)	C6—N2—Mn1	126.9 (2)
C11—C12—H12	119.5	C27—N3—C23	117.8 (6)
C13—C12—H12	119.5	C32—N4—C30	117.6 (5)
C14—C13—C12	120.1 (4)	C32—N4—H1N	120 (4)
C14—C13—H13	120	C30—N4—H1N	122 (4)
C12—C13—H13	120	C41—N5—C40	118.8 (6)
C15—C14—C13	119.2 (4)	C41—N5—H1N	123 (4)
C15—C14—S1	121.2 (3)	C40—N5—H1N	118 (4)
C13—C14—S1	119.6 (3)	C37—N6—C33	116.3 (6)
C14—C15—C16	120.7 (4)	S2—O6—H6	115 (3)
C14—C15—H15	119.7	S2—O6—H6B	98 (2)
C16—C15—H15	119.7	S2—O6B—H6	142 (5)
C11—C16—C15	120.5 (4)	S2—O6B—H6B	116 (3)
C11—C16—H16	119.7	Mn1—O7—H7A	116.4
C15—C16—H16	119.7	Mn1—O7—H7B	109.2
C22—C17—C18	119.2 (3)	H7A—O7—H7B	110.6

C22—C17—C10	118.9 (3)	O1—S1—O2	111.6 (2)
C18—C17—C10	121.9 (3)	O1—S1—O3	113.68 (18)
C19—C18—C17	120.3 (4)	O2—S1—O3	112.49 (18)
C19—C18—H18	119.9	O1—S1—C14	106.19 (19)
C17—C18—H18	119.9	O2—S1—C14	106.04 (17)
C20—C19—C18	119.8 (4)	O3—S1—C14	106.21 (17)
C20—C19—H19	120.1	O6B—S2—O4	101.7 (19)
C18—C19—H19	120.1	O4—S2—O6	112.7 (18)
C19—C20—C21	120.8 (4)	O6B—S2—O5B	111.0 (14)
C19—C20—S2	119.6 (3)	O4—S2—O5B	123.1 (16)
C21—C20—S2	119.6 (3)	O6—S2—O5B	100.0 (12)
C20—C21—C22	119.2 (4)	O6B—S2—O4B	113 (2)
C20—C21—H21	120.4	O6—S2—O4B	122.5 (18)
C22—C21—H21	120.4	O5B—S2—O4B	109.6 (16)
C17—C22—C21	120.8 (4)	O6B—S2—O5	123.4 (12)
C17—C22—H22	119.6	O4—S2—O5	115.9 (15)
C21—C22—H22	119.6	O6—S2—O5	112.4 (10)
N3—C23—C24	122.1 (6)	O4B—S2—O5	102.0 (15)
N3—C23—H23	118.9	O6B—S2—C20	106.1 (8)
C24—C23—H23	118.9	O4—S2—C20	103.7 (16)
C23—C24—C25	121.6 (6)	O6—S2—C20	106.7 (6)
C23—C24—H24	119.2	O5B—S2—C20	109.8 (8)
C25—C24—H24	119.2	O4B—S2—C20	107.6 (16)
C24—C25—C26	114.0 (6)	O5—S2—C20	104.2 (6)
C24—C25—C28	122.3 (5)	N2—Mn1—N2^{i}	180.00 (9)
C26—C25—C28	123.6 (4)	N2—Mn1—N1	90.43 (12)
C27—C26—C25	121.6 (5)	N2^{i}—Mn1—N1	89.57 (12)
C27—C26—H26	119.2	N2—Mn1—N1^{i}	89.57 (12)
C25—C26—H26	119.2	N2^{i}—Mn1—N1^{i}	90.43 (12)
N3—C27—C26	122.8 (6)	N1—Mn1—N1^{i}	180.00 (18)
N3—C27—H27	118.6	N2—Mn1—O7	90.10 (11)
C26—C27—H27	118.6	N2^{i}—Mn1—O7	89.90 (11)
C29—C28—C31	116.3 (5)	N1—Mn1—O7	90.98 (11)
C29—C28—C25	121.4 (4)	N1^{i}—Mn1—O7	89.02 (11)
C31—C28—C25	122.4 (5)	N2—Mn1—O7^{i}	89.90 (11)
C30—C29—C28	120.1 (5)	N2^{i}—Mn1—O7^{i}	90.10 (11)
C30—C29—H29	120	N1—Mn1—O7^{i}	89.02 (11)
C28—C29—H29	120	N1^{i}—Mn1—O7^{i}	90.98 (11)
N4—C30—C29	122.8 (6)	O7—Mn1—O7^{i}	180

N4—C30—H30	118.6	H8A—O8—H8B	108.2
C29—C30—H30	118.6	H9A—O9—H9B	107.5
C32—C31—C28	120.5 (5)	H10A—O10—H10B	108.9
C32—C31—H31	119.7	H11A—O11—H11B	111
C28—C31—H31	119.7	H12B—O12—H12A	109.8
N4—C32—C31	122.7 (5)	H14A—O13—H16B	96.2
N4—C32—H32	118.6	H14A—O14—H14B	112.4
C31—C32—H32	118.6	H16A—O16—H16B	108.4
N6—C33—C34	123.5 (6)		

Symmetry code: (i) −x+2, −y, −z+2.

Table S2 Fractional atomic coordinates and isotropic or equivalent isotropic displacement parameters (Å^2) for compound **1**.

**Atoms**	***x***	***y***	***z***	U_{iso}*/U_{eq}	**Occ. (<1)**
**C1**	0.9495 (4)	0.0089 (4)	0.8643 (2)	0.0273 (8)	
**C2**	0.8818 (4)	0.1008 (4)	0.8172 (2)	0.0316 (8)	
**H2**	0.8754	0.0795	0.7762	0.038*	
**C3**	0.8290 (4)	0.2230 (4)	0.8420 (2)	0.0324 (8)	
**H3**	0.7784	0.3037	0.8217	0.039*	
**C4**	0.8633 (4)	0.2087 (4)	0.9048 (2)	0.0273 (8)	
**C5**	0.8294 (4)	0.3134 (4)	0.9451 (2)	0.0274 (8)	
**C6**	0.8582 (4)	0.3003 (4)	1.0065 (2)	0.0274 (7)	
**C7**	0.8193 (4)	0.4074 (4)	1.0485 (2)	0.0305 (8)	
**H7**	0.7759	0.4984	1.039	0.037*	
**C8**	0.8555 (5)	0.3562 (4)	1.1041 (2)	0.0306 (8)	
**H8**	0.8408	0.4044	1.141	0.037*	
**C9**	0.9204 (4)	0.2152 (3)	1.0972 (2)	0.0256 (7)	
**C10**	1.0239 (4)	−0.1277 (4)	0.8554 (2)	0.0273 (8)	
**C11**	0.7547 (4)	0.4501 (3)	0.9207 (2)	0.0282 (8)	
**C12**	0.8397 (6)	0.5117 (6)	0.8944 (3)	0.079 (2)	
**H12**	0.9484	0.4683	0.8926	0.094*	
**C13**	0.7704 (6)	0.6359 (6)	0.8703 (4)	0.083 (2)	
**H13**	0.8314	0.6771	0.852	0.099*	
**C14**	0.6130 (4)	0.7001 (3)	0.8728 (2)	0.0264 (7)	
**C15**	0.5276 (5)	0.6409 (4)	0.9002 (2)	0.045 (1)	
**H15**	0.4188	0.6856	0.9032	0.053*	
**C16**	0.5973 (5)	0.5159 (4)	0.9239 (2)	0.044 (1)	

**H16**	0.5361	0.4752	0.9425	0.053*	
**C17**	1.0508 (4)	−0.1838 (3)	0.7917 (2)	0.0279 (8)	
**C18**	0.9326 (5)	−0.1859 (4)	0.7619 (2)	0.0338 (9)	
**H18**	0.8309	−0.1498	0.7818	0.041*	
**C19**	0.9626 (5)	−0.2407 (4)	0.7030 (2)	0.039 (1)	
**H19**	0.8815	−0.2419	0.6826	0.047*	
**C20**	1.1103 (5)	−0.2934 (4)	0.6742 (2)	0.0361 (9)	
**C21**	1.2292 (5)	−0.2910 (4)	0.7029 (2)	0.0373 (9)	
**H21**	1.3304	−0.326	0.6826	0.045*	
**C22**	1.1987 (5)	−0.2366 (4)	0.7611 (2)	0.0337 (9)	
**H22**	1.28	−0.2357	0.7822	0.04*	
**C23**	−0.176 (1)	−0.4032 (7)	0.2379 (3)	0.114 (4)	
**H23**	−0.1813	−0.4854	0.2407	0.137*	
**C24**	−0.100 (1)	−0.3722 (6)	0.2801 (3)	0.098 (3)	
**H24**	−0.0515	−0.4351	0.3099	0.118*	
**C25**	−0.0941 (6)	−0.2517 (4)	0.2801 (2)	0.048 (1)	
**C26**	−0.1697 (6)	−0.1681 (5)	0.2344 (3)	0.068 (2)	
**H26**	−0.1754	−0.0816	0.2327	0.082*	
**C27**	−0.2355 (6)	−0.2069 (5)	0.1923 (3)	0.065 (2)	
**H27**	−0.2802	−0.1484	0.1603	0.078*	
**C28**	−0.0207 (6)	−0.2142 (4)	0.3270 (2)	0.047 (1)	
**C29**	0.0774 (6)	−0.3079 (5)	0.3644 (3)	0.059 (1)	
**H29**	0.0998	−0.3982	0.3595	0.071*	
**C30**	0.1410 (7)	−0.2700 (5)	0.4081 (3)	0.064 (1)	
**H30**	0.2063	−0.3353	0.4335	0.077*	
**C31**	−0.0454 (7)	−0.0836 (4)	0.3366 (2)	0.056 (1)	
**H31**	−0.1103	−0.016	0.312	0.067*	
**C32**	0.0224 (7)	−0.0532 (5)	0.3805 (2)	0.059 (2)	
**H32**	0.0034	0.0363	0.386	0.07*	
**C33**	0.4985 (7)	0.2878 (6)	0.6435 (3)	0.069 (2)	
**H33**	0.4908	0.3737	0.6354	0.084*	
**C34**	0.4365 (7)	0.2345 (5)	0.6048 (3)	0.069 (2)	
**H34**	0.3868	0.2843	0.5713	0.083*	
**C35**	0.4457 (5)	0.1098 (5)	0.6140 (2)	0.049 (1)	
**C36**	0.5189 (7)	0.0452 (6)	0.6649 (3)	0.066 (2)	
**H36**	0.5298	−0.0414	0.6736	0.079*	
**C37**	0.5753 (7)	0.1059 (6)	0.7024 (3)	0.070 (2)	
**H37**	0.6209	0.0603	0.7374	0.084*	

**C38**	0.3791 (5)	0.0507 (5)	0.5743 (2)	0.052 (1)	
**C39**	0.2766 (6)	0.1258 (5)	0.5332 (2)	0.056 (1)	
**H39**	0.2497	0.2175	0.5304	0.067*	
**C40**	0.2146 (7)	0.0699 (6)	0.4970 (3)	0.061 (1)	
**H40**	0.1454	0.1235	0.4692	0.073*	
**C41**	0.3478 (7)	−0.1349 (6)	0.5378 (3)	0.070 (2)	
**H41**	0.373	−0.2264	0.5393	0.084*	
**C42**	0.4138 (6)	−0.0842 (5)	0.5750 (3)	0.062 (1)	
**H42**	0.4841	−0.1406	0.6017	0.074*	
**N1**	0.9354 (3)	0.0765 (3)	0.9179 (1)	0.0255 (6)	
**N2**	0.9225 (3)	0.1823 (3)	1.0367 (1)	0.0245 (6)	
**N3**	−0.2400 (6)	−0.3224 (4)	0.1937 (2)	0.0650(1)	
**N4**	0.1154 (5)	−0.1440 (4)	0.4167 (2)	0.056 (1)	
**N5**	0.2484 (5)	−0.0594 (5)	0.4993 (2)	0.0601 (1)	
**N6**	0.5694 (5)	0.2248 (5)	0.6921 (2)	0.0619 (1)	
**O1**	0.5987 (4)	0.9340 (3)	0.8599 (2)	0.0468 (8)	
**O2**	0.5578 (4)	0.8331 (3)	0.7729 (1)	0.0438 (8)	
**O3**	0.3623 (3)	0.9093 (3)	0.8595 (1)	0.0353 (6)	
**O4**	1.073 (4)	−0.243 (4)	0.5621 (2)	0.043 (6)	0.5
**O5**	1.314 (2)	−0.423 (2)	0.5860 (7)	0.046 (3)	0.5
**O6**	1.079 (2)	−0.447 (1)	0.5964 (6)	0.057 (4)	0.5
**O4B**	1.109 (4)	−0.245 (4)	0.5559 (2)	0.042 (6)	0.5
**O5B**	1.306 (3)	−0.447 (2)	0.5844 (9)	0.117 (1)	0.5
**O6B**	1.052 (3)	−0.422 (2)	0.5937 (9)	0.111 (8)	0.5
**O7**	1.2327 (3)	−0.0046 (3)	0.9760 (1)	0.0311 (6)	
**H7A**	1.2745	−0.032	0.9413	0.047*	
**H7B**	1.2277	0.0686	0.9823	0.047*	
**S1**	0.5257 (1)	0.85690 (8)	0.8389 (4)	0.0294 (2)	
**S2**	1.1467 (2)	−0.3568 (1)	0.5972 (4)	0.0442 (3)	
**Mn1**	1	0	1	0.0227 (2)	
**O8**	0.5535 (3)	1.1354 (2)	0.9399 (1)	0.0447 (7)	
**H8A**	0.6146	1.1074	0.9659	0.067*	
**H8B**	0.5616	1.0721	0.9191	0.067*	
**O9**	−0.4426 (4)	−0.3526 (3)	0.1307 (2)	0.074 (1)	
**H9A**	−0.3825	−0.3419	0.1517	0.111*	
**H9B**	−0.4814	−0.2857	0.1099	0.111*	
**O10**	0.5546 (2)	0.4314 (2)	0.76702 (9)	0.088 (2)	
**H10A**	0.5831	0.4823	0.7476	0.132*	

**H10B**	0.5819	0.3633	0.7461	0.132*	
**O11**	0.2477 (3)	1.2320 (2)	0.9845 (1)	0.159 (4)	
**H11A**	0.3407	1.1949	0.9861	0.239*	
**H11B**	0.2276	1.2548	0.9493	0.239*	
**O12**	0.6479 (2)	0.6008 (2)	0.70792 (9)	0.130 (3)	
**H12B**	0.6624	0.6257	0.6732	0.195*	
**H12A**	0.6128	0.6637	0.7324	0.195*	
**O13**	0.6131 (4)	−0.3953 (2)	0.5942 (2)	0.107 (3)*	0.5
**H13B**	0.6391	−0.3935	0.5573	0.16*	0.5
**H13A**	0.656	−0.363	0.6145	0.16*	0.5
**O15**	0.8695 (3)	−0.4201 (2)	0.5154 (2)	0.077 (2)*	0.5
**H15A**	0.7977	−0.4326	0.5049	0.115*	0.5
**H15B**	0.9425	−0.4495	0.4884	0.115*	0.5
**O14**	0.7365 (4)	−0.3399 (2)	0.5915 (2)	0.070 (2)*	0.5
**H14A**	0.7218	−0.3823	0.5655	0.106*	0.5
**H14B**	0.8103	−0.3234	0.5808	0.106*	0.5
**O16**	0.5871 (4)	−0.4566 (2)	0.5298 (2)	0.092 (3)*	0.5
**H16A**	0.5231	−0.4781	0.5171	0.137*	0.5
**H16B**	0.6257	−0.5054	0.5581	0.137*	0.5
**H6**	0.985 (1)	−0.412 (7)	0.589 (2)	0.137*	0.5
**H6B**	0.958 (9)	−0.373 (5)	0.590 (2)	0.137*	0.5
**H1N**	0.193 (1)	−0.107 (9)	0.461 (4)	0.137*	

$$U_{eq} = \frac{1}{3}[U_{11}(aa^*)^2 + U_{22}(bb^*)^2 + U_{33}(cc^*)^2 + 2U_{12}aba^*\,b^*\cos\gamma + 2U_{13}aca^*\,c^*\cos\beta + 2U_{23}bcb^*\,c^*\cos\alpha$$

$U_{iso} = \exp[-8\pi^2 U(\sin^{\theta}/\lambda)^2]$

Table S3 Anisotropic displacement parameters (A^2) for compound **1**.

Atoms	U^{11}	U^{22}	U^{33}	U^{12}	U^{13}	U^{23}
**C1**	0.0238 (18)	0.0307 (18)	0.0208 (16)	−0.0060 (15)	−0.0018 (13)	0.0080 (14)
**C2**	0.031 (2)	0.0320 (19)	0.0196 (16)	−0.0026 (16)	−0.0024 (14)	0.0079 (14)
**C3**	0.032 (2)	0.0309 (19)	0.0230 (17)	−0.0035 (16)	−0.0028 (14)	0.0103 (15)
**C4**	0.0257 (18)	0.0284 (18)	0.0206 (17)	−0.0061 (15)	0.0006 (13)	0.0098 (14)
**C5**	0.0246 (18)	0.0254 (17)	0.0245 (17)	−0.0047 (14)	0.0009 (13)	0.0100 (14)
**C6**	0.0215 (17)	0.0279 (18)	0.0275 (18)	−0.0071 (14)	0.0021 (13)	0.0061 (14)
**C7**	0.031 (2)	0.0233 (17)	0.0320 (19)	−0.0068 (15)	−0.0070 (15)	0.0102 (14)
**C8**	0.037 (2)	0.0283 (18)	0.0251 (18)	−0.0127 (16)	−0.0032 (15)	0.0026 (14)
**C9**	0.0228 (17)	0.0274 (18)	0.0233 (17)	−0.0086 (14)	0.0008 (13)	0.0051 (14)

**C10**	0.0254 (18)	0.0298 (18)	0.0210 (16)	−0.0073 (15)	−0.0012 (13)	0.0056 (14)
**C11**	0.0286 (19)	0.0242 (18)	0.0244 (17)	−0.0051 (15)	−0.0012 (14)	0.0049 (14)
**C12**	0.025 (2)	0.061 (3)	0.138 (6)	−0.010 (2)	−0.010 (3)	0.066 (4)
**C13**	0.030 (3)	0.059 (3)	0.146 (6)	−0.014 (2)	0.001 (3)	0.065 (4)
**C14**	0.0280 (18)	0.0222 (16)	0.0220 (16)	−0.0046 (14)	−0.0034 (13)	0.0075 (13)
**C15**	0.025 (2)	0.033 (2)	0.068 (3)	−0.0065 (17)	−0.0080 (19)	0.022 (2)
**C16**	0.027 (2)	0.035 (2)	0.069 (3)	−0.0133 (18)	−0.0087 (19)	0.025 (2)
**C17**	0.032 (2)	0.0245 (17)	0.0215 (17)	−0.0069 (15)	−0.0039 (14)	0.0075 (14)
**C18**	0.036 (2)	0.034 (2)	0.0290 (19)	−0.0131 (17)	−0.0039 (16)	0.0085 (16)
**C19**	0.056 (3)	0.035 (2)	0.032 (2)	−0.022 (2)	−0.0188 (19)	0.0110 (17)
**C20**	0.058 (3)	0.0247 (18)	0.0203 (17)	−0.0132 (18)	−0.0058 (17)	0.0061 (14)
**C21**	0.046 (2)	0.031 (2)	0.0271 (19)	−0.0097 (18)	−0.0003 (17)	0.0037 (15)
**C22**	0.037 (2)	0.033 (2)	0.0235 (18)	−0.0082 (17)	−0.0024 (15)	0.0005 (15)
**C23**	0.230 (11)	0.047 (3)	0.065 (4)	−0.054 (5)	−0.048 (5)	0.018 (3)
**C24**	0.200 (9)	0.037 (3)	0.052 (3)	−0.040 (4)	−0.038 (4)	0.019 (2)
**C25**	0.048 (3)	0.032 (2)	0.046 (2)	−0.0062 (19)	0.019 (2)	0.0083 (18)
**C26**	0.038 (3)	0.032 (2)	0.128 (6)	−0.008 (2)	−0.020 (3)	0.022 (3)
**C27**	0.046 (3)	0.040 (3)	0.106 (5)	−0.015 (2)	−0.014 (3)	0.022 (3)
**C28**	0.047 (3)	0.029 (2)	0.051 (3)	−0.0073 (19)	0.020 (2)	0.0051 (19)
**C29**	0.055 (3)	0.029 (2)	0.079 (4)	−0.008 (2)	0.006 (3)	−0.002 (2)
**C30**	0.057 (3)	0.037 (3)	0.081 (4)	−0.004 (2)	−0.001 (3)	−0.001 (3)
**C31**	0.079 (4)	0.030 (2)	0.039 (2)	−0.012 (2)	0.024 (2)	0.0041 (19)
**C32**	0.084 (4)	0.034 (2)	0.045 (3)	−0.019 (3)	0.023 (3)	0.001 (2)
**C33**	0.068 (4)	0.044 (3)	0.076 (4)	−0.003 (3)	−0.015 (3)	0.020 (3)
**C34**	0.072 (4)	0.047 (3)	0.069 (4)	−0.004 (3)	−0.024 (3)	0.023 (3)
**C35**	0.030 (2)	0.043 (2)	0.055 (3)	0.0002 (19)	0.008 (2)	0.021 (2)
**C36**	0.059 (3)	0.057 (3)	0.078 (4)	−0.022 (3)	−0.012 (3)	0.035 (3)
**C37**	0.064 (4)	0.070 (4)	0.076 (4)	−0.027 (3)	−0.020 (3)	0.040 (3)
**C38**	0.032 (2)	0.051 (3)	0.051 (3)	−0.002 (2)	0.012 (2)	0.015 (2)
**C39**	0.047 (3)	0.051 (3)	0.055 (3)	−0.011 (2)	0.003 (2)	0.016 (2)
**C40**	0.055 (3)	0.062 (3)	0.051 (3)	−0.014 (3)	0.006 (2)	0.011 (3)
**C41**	0.056 (3)	0.054 (3)	0.079 (4)	−0.006 (3)	0.003 (3)	0.001 (3)
**C42**	0.048 (3)	0.049 (3)	0.069 (3)	−0.004 (2)	−0.001 (3)	0.011 (3)
**N1**	0.0218 (14)	0.0258 (15)	0.0196 (14)	−0.0026 (12)	0.0008 (11)	0.0056 (11)
**N2**	0.0247 (15)	0.0226 (14)	0.0186 (14)	−0.0039 (12)	0.0003 (11)	0.0064 (11)
**N3**	0.079 (3)	0.048 (2)	0.064 (3)	−0.026 (2)	0.001 (2)	0.011 (2)
**N4**	0.058 (3)	0.041 (2)	0.056 (2)	−0.016 (2)	0.018 (2)	−0.0080 (19)
**N5**	0.048 (2)	0.060 (3)	0.056 (3)	−0.012 (2)	0.009 (2)	−0.002 (2)
**N6**	0.046 (2)	0.060 (3)	0.068 (3)	−0.012 (2)	−0.008 (2)	0.021 (2)

**O1**	0.0454 (18)	0.0292 (15)	0.065 (2)	−0.0161 (13)	0.0007 (15)	−0.0043 (14)
**O2**	0.0521 (18)	0.0367 (15)	0.0225 (13)	−0.0022 (14)	0.0023 (12)	0.0076 (11)
**O3**	0.0346 (15)	0.0275 (13)	0.0316 (14)	−0.0033 (11)	0.0011 (11)	0.0073 (11)
**O4**	0.062 (14)	0.038 (6)	0.026 (11)	−0.019 (8)	−0.006 (9)	0.001 (6)
**O5**	0.062 (6)	0.040 (5)	0.019 (4)	−0.007 (5)	0.008 (4)	−0.015 (3)
**O6**	0.132 (11)	0.024 (3)	0.030 (5)	−0.042 (4)	−0.027 (6)	0.002 (3)
**O4B**	0.064 (15)	0.030 (5)	0.018 (4)	−0.010 (8)	0.005 (8)	0.008 (3)
**O5B**	0.137 (15)	0.075 (12)	0.053 (8)	0.047 (9)	−0.051 (8)	−0.023 (7)
**O6B**	0.157 (16)	0.19 (2)	0.055 (10)	−0.145 (17)	0.013 (10)	−0.012 (12)
**O7**	0.0302 (14)	0.0332 (14)	0.0233 (12)	−0.0092 (12)	0.0041 (10)	0.0014 (10)
**S1**	0.0335 (5)	0.0205 (4)	0.0247 (4)	−0.0043 (4)	0.0020 (3)	0.0046 (3)
**S2**	0.0720 (8)	0.0315 (5)	0.0242 (5)	−0.0167 (5)	−0.0102 (5)	0.0031 (4)
**Mn1**	0.0220 (4)	0.0212 (4)	0.0170 (4)	−0.0023 (3)	−0.0010 (3)	0.0069 (3)
**O8**	0.0351 (16)	0.0542 (19)	0.0417 (16)	−0.0150 (14)	−0.0092 (12)	−0.0032 (14)
**O9**	0.063 (3)	0.053 (2)	0.100 (3)	−0.019 (2)	−0.006 (2)	−0.005 (2)
**O10**	0.119 (4)	0.072 (3)	0.070 (3)	−0.031 (3)	−0.048 (3)	0.027 (2)
**O11**	0.064 (3)	0.123 (5)	0.296 (9)	−0.061 (3)	0.081 (4)	−0.128 (6)
**O12**	0.197 (7)	0.125 (5)	0.095 (4)	−0.107 (5)	0.061 (4)	−0.061 (4)

U_{ij} = exp (-2π^2[h^2(a*)^{2}U$_{11}$ + k^2(b*)^{2}U$_{22}$ + l^2(c*)^{2}U$_{33}$ + 2hka*b*U$_{12}$ 2hla*c*U$_{13}$ + 2klb*c*U$_{23}$])

Table S4 Hydrogen bond parameters for compound **1** (distances in Å and angles in º).

O-H	A(O)	O-H (Å)	H···A (Å)	O···A (Å)	O-H···A (º)
O(7)-H(7A)	O(3) *(-1+x,1+y,z)*	0.82	1.90	2.723(4)	178
O(7)-H(7B)	O(11) *(-1+x,y,z)*	0.82	1.94	2.740(4)	167
O(8)-H(8A)	O(7) *(-x,2-y,-z)*	0.82	2.00	2.809(4)	169
O(11)-H(11A)	O(8)	0.82	2.03	2.759(4)	147
O(9)-H(9A)	N(3) *(1-x,1-y,1-z)*	0.83	1.83	2.657(7)	176
O(9)-H(9B)	O(8)	0.83	1.89	2.720(4)	175
O(10)-H(10B)	N(6) *(1-x,1-y,1-z)*	0.83	2.03	2.823(5)	160
O(10)-H(10A)	O(12)	0.83	1.87	2.692(3)	177
O(12)-H(12A)	O(13)	0.82	1.83	2.540(4)	145
O(12)-H(12A)	O(14)	0.82	1.97	2.766(4)	163
O(13)-H(13A)	O(12)	0.83	2.07	2.540(4)	123
O(13)-H(13B)	O(15)	0.82	2.22	2.787(5)	126
O(15)-H(15A)	O(13)	0.82	2.42	2.787(5)	108
O(15)-H(15A)	O(16)	0.82	2.19	2.936(5)	150

O(12)-H(12B)	O(2)	0.82	1.94	2.752(4)	169
O(14)-H(14B)	O(6) *(x,-1+y,z)*	0.82	2.28	3.010(2)	148
O(14)-H(14B)	O(6B) *(x,-1+y,z)*	0.82	2.01	2.770(3)	153
O(16)-H(16A)	O(5)	0.83	2.26	2.667(2)	111
O(16)-H(16A)	O(5B) *(1+x,-1+y,z)*	0.83	2.35	2.820(3)	117
O(6)-H(6)	O(15) *(x,1+y,z)*	0.86	2.35	2.745(2)	109
O(6)-H(6B)	O(14) *(x,1+y,z)*	1.15	1.99	3.010(2)	145
O(6)-H(6B)	O(15) *(x,1+y,z)*	1.15	2.16	2.745(2)	108
N(5)-H(1N)	N(4)	1.28	1.46	2.741(7)	171

Table S5 Substrate overview for all the catalytic reactions.

**Reaction**	**Substrate**
Oxidation of alcohols	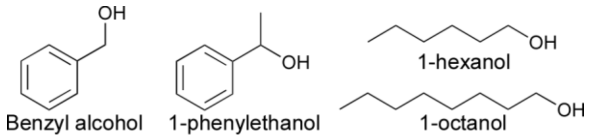
Aldol condensations	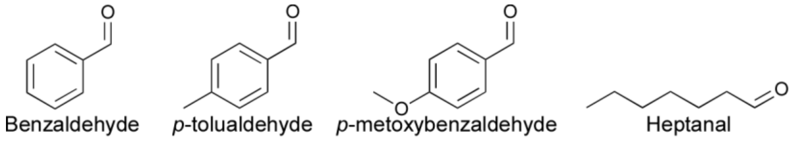
Knoevenagel condensations	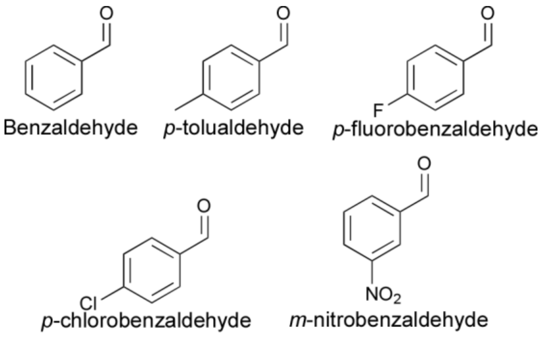
One-pot cascade (acetal hydrolysis + Knoevenagel condensation	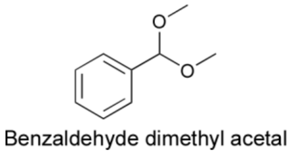

Table S6 Recyclability of the compound **1** and **3** for benzyl alcohol oxidation.

Compound **1**		Compound **3**	
**Cycles**	**C_T (4h)**	**Cycles**	**C_T (4h)**
1	40%	1	27.31%
2	31.3%	2	31.42%
3	30.69%	3	32%
4	26.8%	4	26.8%
5	26.4%	5	26%

C_T = Total conversion

Table S7 Recyclability of the compound **2** for benzaldehyde condensation.

Compound **2**	
**Cycles**	**C_T (4h)**
1	34.46%
2	33.07%
3	33.95%
4	25.60%
5	21.03%
